# Long-Term Field Performance of Sensirion SEN54 and SPS30 Low-Cost Particle Sensors: Seasonal Variability and Correction Using Dew Point Deficit

**DOI:** 10.3390/s26216914

**Published:** 2026-10-31

**Authors:** Petra Tesařová, Adriana Šindelářová, Josef Keder, Petr Vincenc, Štěpán Rychlík

**Affiliations:** Czech Hydrometeorological Institute, Na Šabatce 2050/17, 143 06 Prague 4, Czech Republic; adriana.sindelarova@chmi.cz (A.Š.); josef.keder@chmi.cz (J.K.); petr.vincenc@chmi.cz (P.V.); stepan.rychlik@chmi.cz (Š.R.)

**Keywords:** optical particle counters, low-cost sensors, dew point deficit, season, MARS

## Abstract

**Highlights:**

- Sensirion SPS30 and SEN54 sensors exhibited pronounced seasonal variability, performing substantially better during the cold season than during the warm season.
- Dew point deficit (DPD) was identified as an effective meteorological predictor, while particle count-based correction further improved PM10 estimates.
- DPD provided a simple and physically interpretable basis for correcting low-cost PM sensor measurements.
- Persistent warm-season underestimation suggests that aerosol properties influence sensor response beyond the effects of meteorological conditions alone.

**Abstract:**

Low-cost optical particle counters (OPCs) are increasingly used for supplementary air-quality monitoring, but their long-term performance remains insufficiently characterised. This study evaluated two pairs of Sensirion OPCs (SEN54 and SPS30) against a Palas Fidas 200S equivalent monitor (EM) over a 19-month field deployment. Sensor agreement was considerably lower for PM_10_ (mean R^2^ = 0.50, mean RMSE = 13.70 µg·m^−3^) than for PM_2.5_ (mean R^2^ = 0.87, mean RMSE = 5.53 µg·m^−3^). None of the sensors adequately captured elevated PM_10_ episodes associated with mineral dust, and removing these episodes moderately improved agreement with the EM. Both sensor types exhibited pronounced seasonal variability, with substantially better performance during the cold season. A large proportion of this variability was explained by atmospheric moisture conditions represented by the dew point deficit (DPD). MARS-based correction models combining sensor-reported PM concentrations and DPD improved performance to mean R^2^ values of 0.68 for PM_10_ (mean RMSE = 8.25 µg·m^−3^) and 0.92 for PM_2.5_ (mean RMSE = 3.29 µg·m^−3^). For SPS30 sensors, particle-count and DPD-based corrections further improved PM_10_ and PM_2.5_ performance, although high PM_10_ episodes remained challenging for some sensors. Residual warm-season underestimation suggests that aerosol-specific properties beyond atmospheric moisture continue to affect sensor accuracy.

## 1. Introduction

In line with growing public awareness of the impacts of air pollution on human health, the demand for low-cost air-quality sensor networks has increased substantially over the past two decades. These technologies offer the potential to improve the spatial and temporal coverage of air-quality monitoring and to provide information even in locations where conventional monitoring infrastructure is unavailable [1,2]. Although the advantages of low-cost sensors, including their low purchase cost, compact size, and ease of deployment, as well as their limitations related to measurement accuracy and sensitivity to environmental conditions, have been widely recognised, continued evaluation remains essential because sensor performance cannot be assumed to remain constant across successive sensor generations. Manufacturers are continuously introducing improvements in both hardware design and proprietary data-processing algorithms, which may alter sensor response characteristics and consequently affect measurement performance under real-world conditions.

One category of sensors that is attracting considerable interest from both the general public and experts is low-cost optical particle counters (OPCs). Low-cost OPCs estimate particulate matter (PM) mass concentrations indirectly from light-scattering measurements and particle-sizing algorithms and not by direct gravimetric determination (as in the case of reference PM measurement). Consequently, sensor performance depends not only on the optical hardware itself but also on the proprietary algorithms converting particle counts and size distributions into reported PM mass concentrations [3,4,5]. Overall, most of the studies and guidelines published to date have reported generally better performance in measuring concentrations of the finer aerosol fractions PM_1_ and PM_2.5_ than in the case of the coarse PM_10_ fraction [6,7,8,9], which is often subject to greater uncertainty and is mostly dependent on the type of aerosol (including particle size, density, morphology and chemical composition), humidity and the sensor’s initial calibration [4,8,10,11].

This study focused on the long-term evaluation of the performance of Sensirion optical particle counters (OPCs), specifically the SEN54 and SPS30 sensor types, under ambient outdoor conditions. Previous studies have generally identified SPS30 as one of the better-performing low-cost OPCs currently available, exhibiting high inter-sensor reproducibility and lower inter-unit variability than several commonly used alternatives, including Plantower, Honeywell and Sharp sensors [12,13,14]. The reported performance has generally been most satisfactory for the fine particle fractions PM_1_ and PM_2.5_, while substantially weaker results have been observed for the coarse PM_10_ fraction [13,14,15,16,17]. The reduced accuracy of PM_10_ measurements is commonly attributed to the limited ability of low-cost optical sensors to characterise coarse particles and to the uncertainties associated with converting particle-size information into PM mass concentrations [4,11,14].

Environmental conditions are well known to influence the performance of low-cost OPCs. Seasonal variability is particularly pronounced in regions with strong climatic contrasts and seasonally changing pollution sources. Consequently, most calibration approaches incorporate temperature and relative humidity, which affect optical PM measurements through hygroscopic particle growth and associated changes in particle optical properties [6,7,15,18]. Because these effects are often non-linear, non-linear regression and machine learning approaches generally outperform simple linear models.

Interestingly, several recent studies have suggested that Sensirion sensors may be less susceptible to direct humidity-related artefacts than many earlier generations of optical particle counters [13,17]. Nevertheless, calibration models incorporating temperature and humidity still have consistently improved agreement with reference instruments, indicating that meteorological conditions remain an important source of variability even when obvious humidity-induced artefacts are largely mitigated [13,15,19]. Under these circumstances, variables that integrate information from both temperature and humidity may provide a more physically interpretable description of atmospheric moisture conditions than treating multiple meteorological variables and their interactions separately. One such variable is the dew point deficit (DPD), which combines temperature and humidity into a single metric describing overall moisture conditions [20]. Low DPD values indicate near-saturated air and favourable conditions for hygroscopic particle growth, whereas high DPD values correspond to drier atmospheric conditions and possible particle shrinkage through water loss.

Apart from meteorological correction approaches, several recent studies have suggested that the use of particle-count information may improve calibration performance compared with models based solely on manufacturer-reported PM mass concentrations [21,22]. In particular, [21] demonstrated that calibration models incorporating particle-count information consistently outperformed models relying exclusively on sensor-reported PM values, suggesting that part of the information relevant for PM estimation may be lost during the internal conversion of particle counts into reported mass concentrations performed by sensor firmware [23]. Therefore, correction approaches that make direct use of particle-count data may provide additional information beyond the PM concentrations reported by the sensor and warrant further investigation.

The performance of low-cost optical PM sensors depends strongly on aerosol properties, including particle size distribution, refractive index and hygroscopicity, and therefore a universal conversion between light scattering and PM mass concentration does not exist [24,25,26]. Consequently, correction factors derived for one location may not be directly transferrable to another environment with different aerosol sources and properties. Site-specific calibration and validation therefore remain strongly recommended [1,24].

This study aimed to (i) identify a simple physically interpretable meteorological predictor most strongly associated with the performance of Sensirion SEN54 and SPS30 sensors, (ii) evaluate the ability of a dew point deficit to explain seasonal variability in sensor response, (iii) develop MARS-based correction models using both sensor-reported PM concentrations and particle-count variables and (iv) assess their performance against an equivalent Fidas monitor. Particular emphasis was placed on determining whether the use of particle-count information can improve PM_10_ estimation compared with conventional mass-based correction approaches.

## 2. Materials and Methods

### 2.1. Study Area and Experimental Design

**Figure 1:**
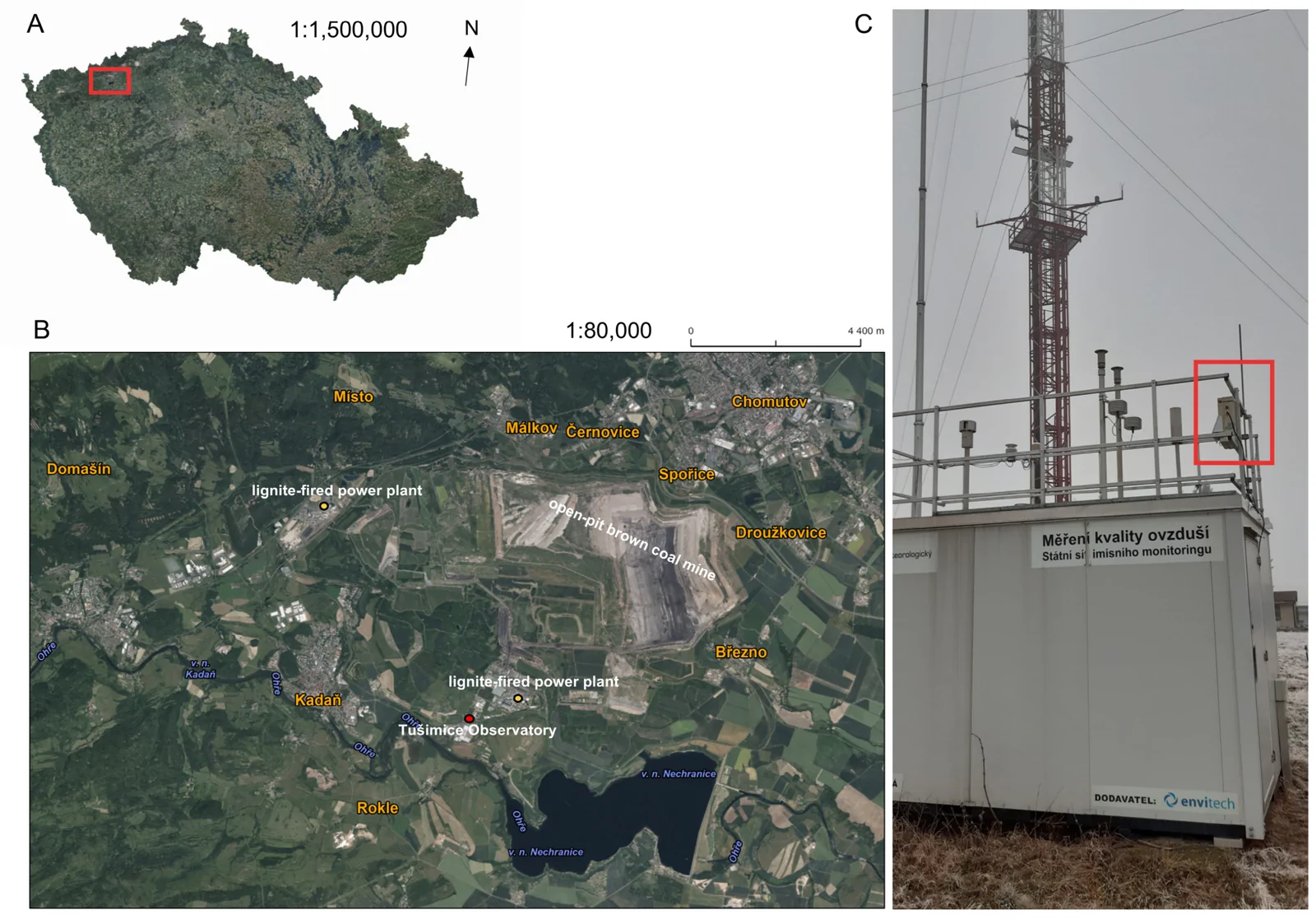
(**A**) Map of the Czech Republic with the position of the Tušimice Observatory. (**B**) Map of Tušimice Observatory and its surroundings. (**C**) A low-cost sensor box equipped with Sensirion SEN54 (A,B) and SPS30 (A,B) particle counters, installed on the roof of the ambient air quality-monitoring station in Tušimice. The orthophoto was provided through WMS by the Czech Office for Surveying, Mapping and Cadastre—ČÚZK.

This study focused on long-term performance testing of two types of low-cost particle counters from Sensirion, specifically two SEN54 units (hereinafter referred to as SEN54A, SEN54B) and two SPS30 units (SPS30A, SPS30B). Both types of particle counters tested were used to measure the mass concentration of aerosols in size fractions PM_10_, PM_4_, PM_2.5_ and PM_1_ [27,28]. According to the technical specifications of both sensor types, PM_4_ and PM_10_ concentrations are derived from measurements of the smaller PM_2.5_ and PM_1_ particles (i.e., the coarse fractions are not measured directly but are calculated) [29]. The SPS30 model also provides particle counts in the 0.5, 1, 2.5, 4 and 10 µm size fractions [28]. The comparative measurements lasted from 1 February 2024 to 31 August 2025 (577 days). All units were installed in appropriate housing (ventilated box) on the roof of the automated ambient air quality-monitoring station at Tušimice Observatory (GPS: 50°22′35.59″ N, 13°19′39.76″ E; station owner CHMI) [30]. The station is classified as a rural background station with the characteristics of an agricultural–industrial zone (nearby are two thermal power plants that burn lignite, as well as one large-scale open-pit lignite mine; see Figure 1). All data measured by the sensors were compared with an equivalent aerosol analyser Palas Fidas 200S [31] (hereinafter referred to as EM Fidas). Furthermore, the hourly mean air temperature and relative humidity data from a co-located professional meteorological station in Tušimice [32] were used as input variables for the correction models and for calculating the dew point deficit (DPD).

### 2.2. Data Processing and Statistical Analysis

All sensor output data were averaged over 1 h before comparison with EM Fidas. To ensure successful averaging, a minimum data completeness threshold of 70% was set. To identify systematic deviations and potential outliers in hourly sensor measurements, the individual measurement bias was calculated as the difference between the concentrations measured by the sensors and those measured by the EM Fidas. In addition, the sensor performance ratio (SPR), defined as the ratio of the sensor to the EM Fidas concentration [20], was calculated and used as a metric of sensor performance relative to EM Fidas. An SPR value of one indicates perfect agreement, values below one indicate underestimation and values above one indicate overestimation by the sensor.

First, the general effect of seasonality on sensor measurement quality was assessed. Two seasons were selected based on the calendar and typical conditions in Central Europe—the cold season covering the months of October through March and the warm season covering April through September (for descriptive statistics of meteorological parameters, see Table S1 in the Supplementary Materials). Seasonal differences in SPR were then evaluated using the Wilcoxon rank-sum test. In addition, Cliff’s delta non-parametric effect size measure was calculated to quantify the magnitude of differences between SPR values in two different seasons (ranging from −1 to 1).

Due to the known effect of relative humidity (RH, %) and temperature (T, °C) on sensor performance, the dew point temperature (T_d_, °C) and dew point deficit (DPD, °C) were calculated according to Equations (1)–(3).

(1) $$A=\ln\left(\frac{\mathrm{RH}}{100}\right)+\frac{17.625\;T}{243.04+T}$$

(2) $$T_{d}=\frac{243.04\;A}{17.625-A}$$

DPD = T − T_d_ (3)

To investigate the influence of seasonality and specific (measured or calculated) meteorological conditions on sensor performance, three regression models were developed using the sensor performance ratio (SPR) as the response variable and different combinations of season, temperature (T), relative humidity (RH), and dew point deficit (DPD) as explanatory variables. The significance of explanatory variables and differences among the candidate regression models were evaluated using analysis of variance (ANOVA). The fit of the tested models was evaluated using the coefficient of determination (R^2^), adjusted coefficient of determination (adjusted R^2^), and Akaike Information Criterion (AIC). While R^2^ and adjusted R^2^ were used to assess the proportion of variability explained by each model and the AIC was used to evaluate model quality while accounting for model complexity, the RMSE was used to quantify the average magnitude of prediction errors. Models exhibiting higher R^2^ and adjusted R^2^ values together with lower AIC and RMSE values were considered to provide a better fit with the observed data. Prior to model testing, DPD values were mean-centred (DPDc = DPD − mean DPD) to reduce collinearity between the linear and quadratic terms included in model M3 (see the Results Section 3.3).

In the final stage, the raw data from the LCS were corrected using the MARS method [1,33], where EM Fidas concentrations served as dependent variable and sensor measurements and T, RH, DPD, season and hour of day were used in various combinations as explanatory variables (see the description of the main correction models CM0–CM4 in Table 1; and the full set of MARS correction models CM0e–CM5e tested in Table S2 in the Supplementary Materials). The MARS corrections were developed with R software (version 4.6.1, 2026) using the earth package [34]. Two scenarios were evaluated. In the first (practical scenario), the training dataset used for the development of the MARS correction models consisted of the first 2 months of observation (1 February to 30 March 2024; 58 days). In the second scenario (the extended-training scenario), the training dataset consisted of the first 6 months of observations (1 February to 31 July 2024; 181 days). The performance of individual MARS models was evaluated using the coefficient of determination (R^2^) and the Generalised Cross-Validation (GCV) error. Subsequently, the derived MARS correction equations were applied to an independent testing dataset comprising the remaining observations (in the practical scenario from 31 March 2024 to 31 August 2025, 518 days; or in the extended-training scenario from 1 August 2024 to 31 August 2025, 395 days). Corrected PM concentrations were then compared with the corresponding EM Fidas measurements. The final model performance was evaluated exclusively on the testing dataset using the coefficient of determination (R^2^), root mean square error (RMSE), mean absolute error (MAE), bias, and the slope and intercept of the linear regression between the corrected sensor concentrations and the EM Fidas measurements.

Finally, based on [21], an alternative correction model using sensor particle-count data and DPD as explanatory variables was tested for both SPS30 sensors (see MC4 in Table 1). The analyses presented in Section 3.1, Section 3.2 and Section 3.3 were based on the complete observation dataset and refer to raw sensor measurements. For all correction models in Section 3.4, model development and performance evaluation were conducted using separate training and independent testing datasets, and final performance statistics were calculated exclusively from the testing datasets.

## 3. Results

### 3.1. Raw Sensor Performance Evaluation

**Figure 2:**
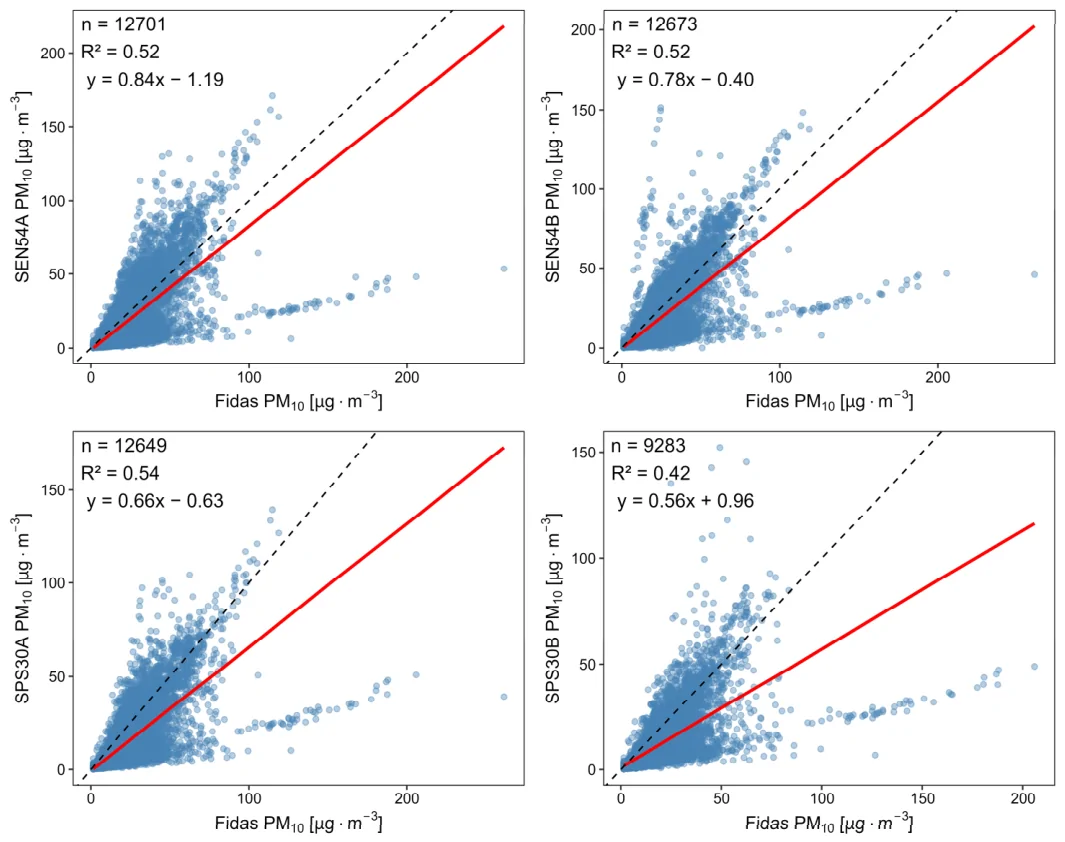
Scatterplots showing the relationship between raw PM_10_ concentrations measured by the four tested Sensirion sensors (SEN54A, SEN54B, SPS30A and SPS30B) and the equivalent-method (EM) Fidas analyser during the entire study period (1 February 2024–31 August 2025). The solid red line represents the linear regression fit, while the dashed black line indicates the 1:1 relationship. Values of n, coefficient of determination (R^2^), and regression equation are shown in each panel.

**Figure 3:**
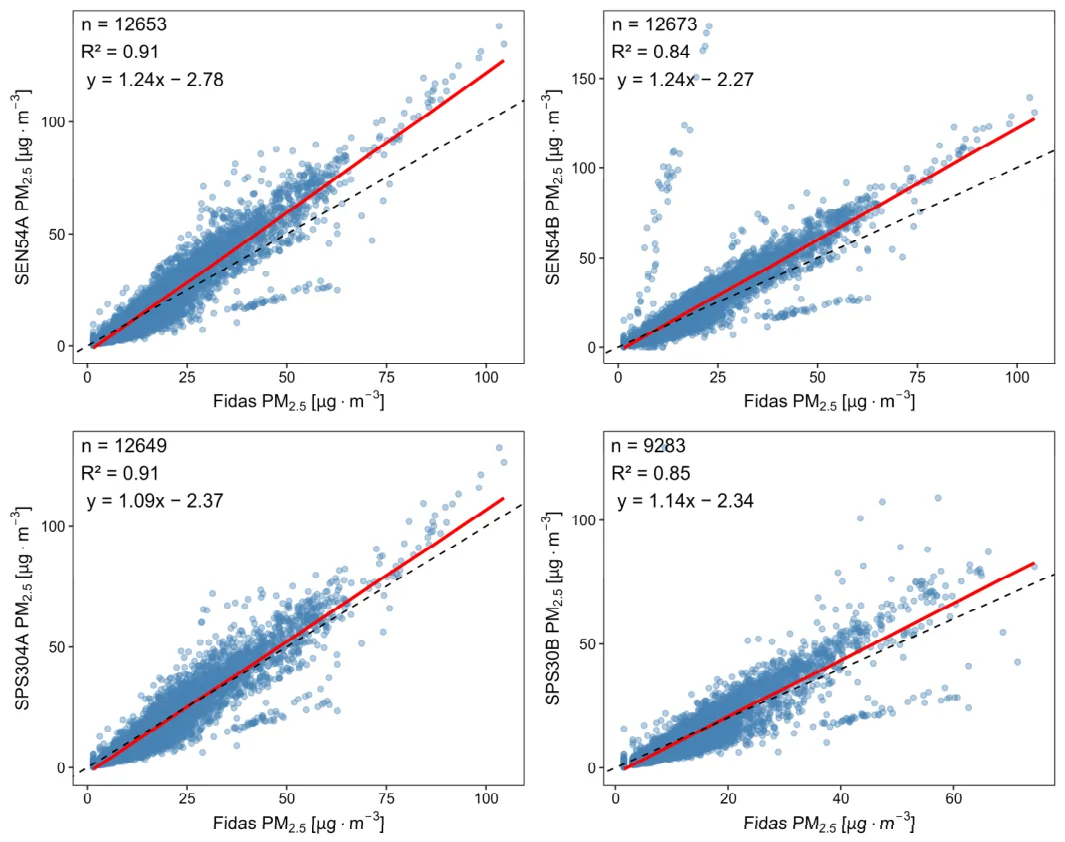
Scatterplots showing the relationship between raw PM_2.5_ concentrations measured by the four tested Sensirion sensors (SEN54A, SEN54B, SPS30A and SPS30B) and the equivalent-method (EM) Fidas analyser during the entire study period (1 February 2024–31 August 2025). The solid red line represents the linear regression fit, while the dashed black line indicates the 1:1 relationship. Values of n, coefficient of determination (R^2^), and the regression equation are shown in each panel.

The descriptive statistics for the PM_10_ and PM_2.5_ concentrations measured by the individual Sensirion sensors and the EM Fidas analyser during the entire study period are presented in Table S3 in the Supplementary Materials. All four tested sensors (SEN54A, SEN54B, SPS30A and SPS30B) showed similarly consistent performances throughout the study, except for SPS30B, which experienced more frequent measurement outages; only 67% of its values were valid, compared with more than 90% for the other sensors. Agreement among the sensor units was very high, with Spearman correlation coefficients of r_S_ > 0.98 for both PM_10_ and PM_2.5_ measurements (Tables S4 and S5 in the Supplementary Materials). Compared with the EM Fidas, substantially better performance was achieved for PM_2.5_ than for PM_10_ measurements. For PM_10_, performance metrics included R^2^ values of 0.42–0.54, RMSE values of 13.51–14.15 µg·m^−3^, and slopes of 0.56–0.84, with all sensors exhibiting a negative bias in relative to EM Fidas. In contrast, PM_2.5_ measurements showed R^2^ values of 0.84–0.91, RMSE values of 4.47–7.18 µg·m^−3^, slopes of 1.09–1.24 and bias values close to zero. Complete performance statistics are presented in Table 2, while the corresponding linear regression plots for PM_10_ and PM_2.5_ are shown in Figure 2 and Figure 3, respectively.

### 3.2. Identification of Specific Episodes with High PM_10_ Concentrations

Several episodes characterised by unusually high concentrations of coarse PM_10_ particles were identified from the EM Fidas measurements (52 hourly observations in total; see Table S6 in the Supplementary Materials). During these episodes, the Sensirion sensors failed to capture the elevated PM_10_ concentrations recorded by the EM Fidas (Figure S1). The corresponding measurement bias exceeded 70 µg·m^−3^, indicating substantial underestimation of PM_10_ concentrations by the sensors (Figures S2 and S3 in the Supplementary Materials). The identified episodes were associated with mineral dust events, including Saharan dust intrusions and resuspension of coarse particles from nearby surface mining activities. Since the PM_10_ concentrations reported by the sensors are derived from internal particle-sizing algorithms rather than direct measurements of coarse particle mass [29], the observed discrepancies were considered an inherent limitation of the sensor technology rather than a measurement malfunction. Therefore, these episodes were excluded from subsequent analyses. Following their removal, the average sensor performance improved to R^2^ = 0.61 and RMSE = 11.64 µg·m^−3^ for PM_10_ measurements, and to R^2^ = 0.90 and RMSE = 5.30 µg·m^−3^ for PM_2.5_ measurements (complete results are provided in Table S7 and Figures S4–S6 in the Supplementary Materials).

### 3.3. Seasonal Differences in Sensor Performance and the Influence of DPD

**Figure 4:**
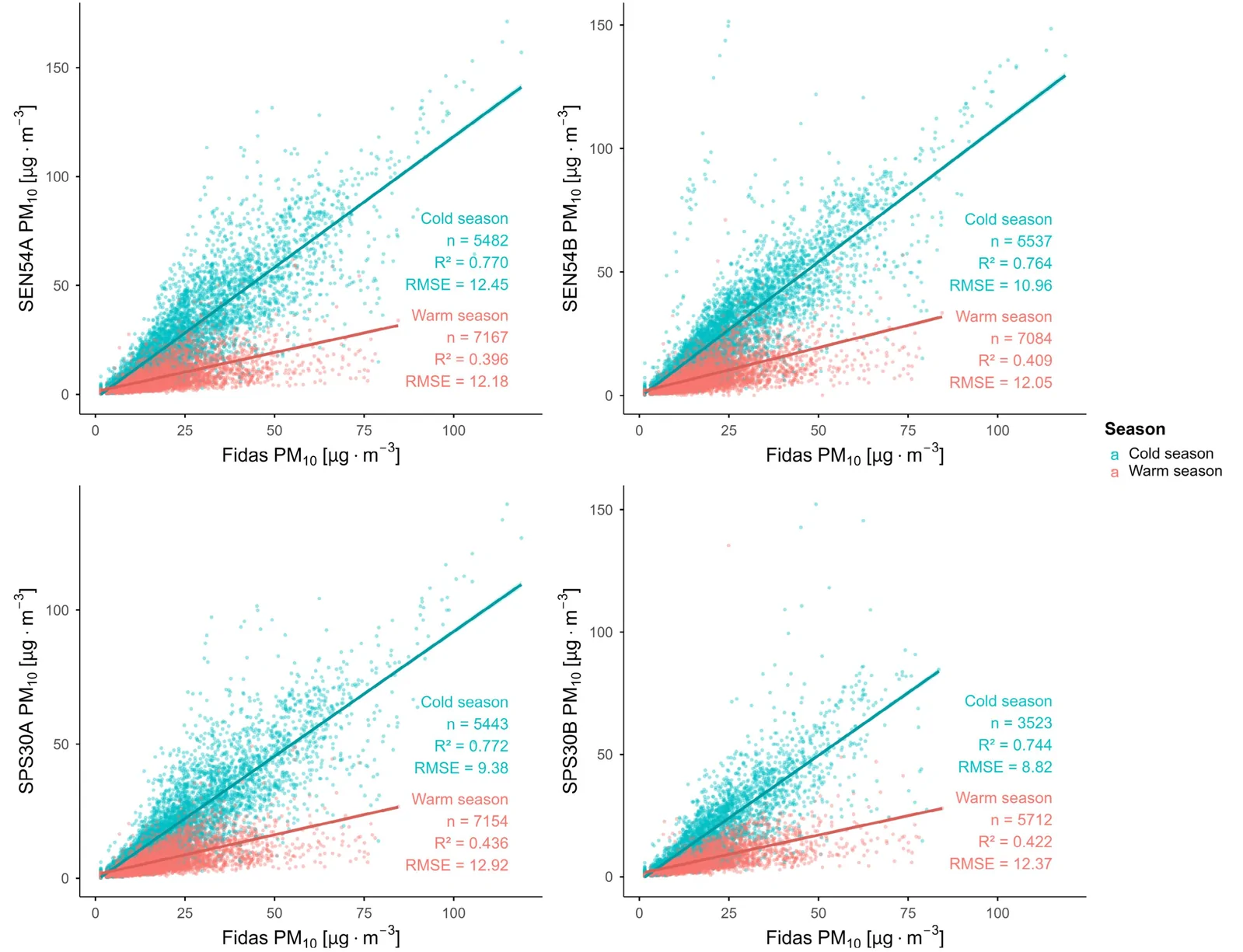
Comparison of PM_10_ concentrations (µg·m^−3^) measured by four low-cost optical sensors (SEN54A, SEN54B, SPS30A and SPS30B) and the EM Fidas equivalent analyser during the cold and warm seasons in Tušimice between 1 February 2024 and 31 August 2025. The solid lines indicate season-specific linear regression fits (blue: cold season; red: warm season). The corresponding number of observations (n), coefficients of determination (R^2^) and root mean square errors (RMSEs) are provided for each season.

**Figure 5:**
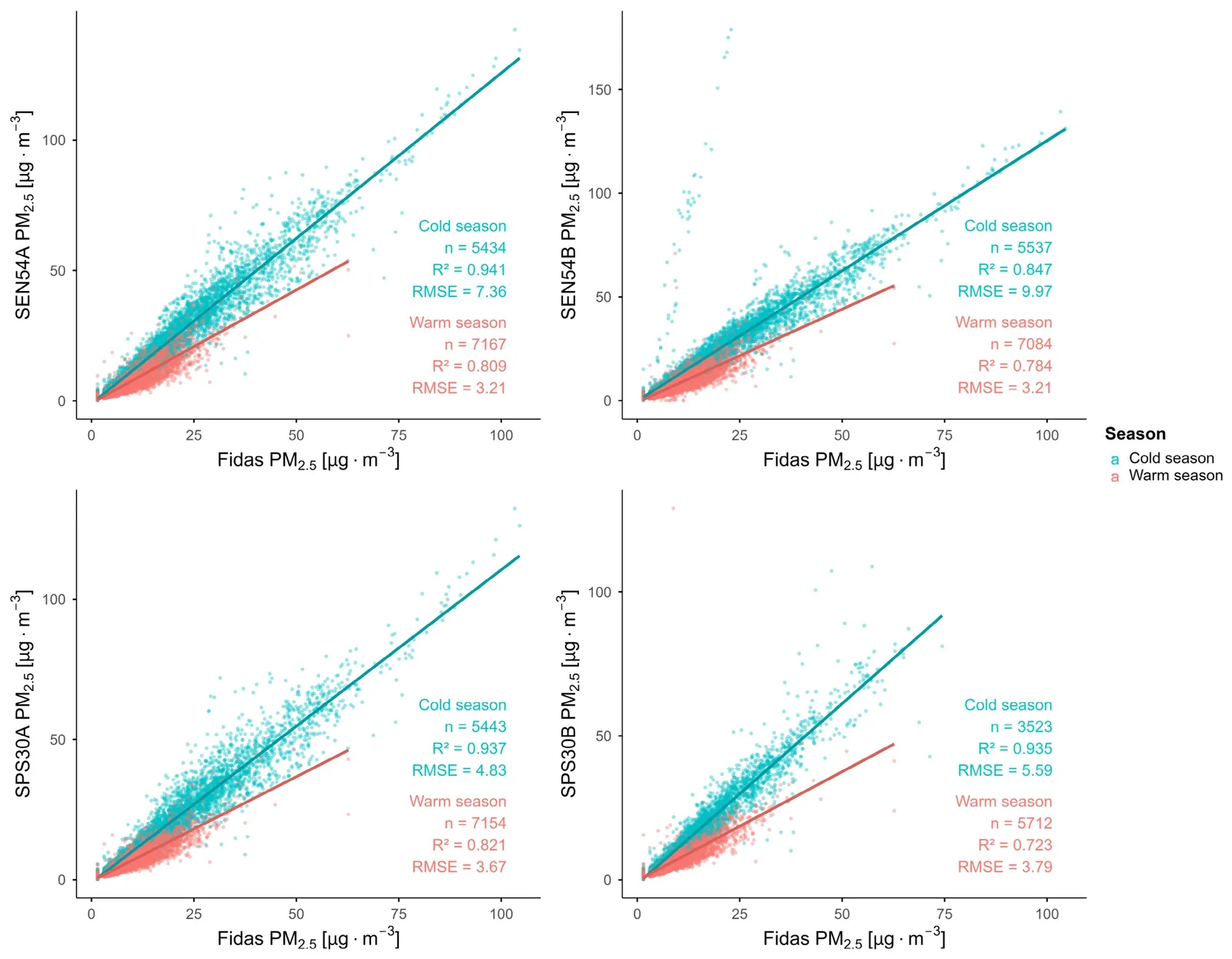
Comparison of PM_2.5_ concentrations (µg·m^−3^) measured by four low-cost optical sensors (SEN54A, SEN54B, SPS30A and SPS30B) and the EM Fidas equivalent analyser during the cold and warm seasons in Tušimice between 1 February 2024 and 31 August 2025. The solid lines indicate season-specific linear regression fits (blue: cold season; red: warm season). The corresponding number of observations (n), coefficients of determination (R^2^) and root mean square errors (RMSEs) are provided for each season.

Monthly distributions of the differences between sensor measurements and EM Fidas concentrations (bias) showed pronounced seasonal patterns for both PM_10_ and PM_2.5_ measurements (Figures S3 and S5 in the Supplementary Materials). Warm-season measurements were characterised by a relatively small spread but systematic underestimation of reference concentrations. In contrast, cold-season measurements showed reduced systematic bias but substantially larger variability and more frequent extreme deviations. Seasonal difference in sensor performance was observed for all the tested sensors (Figure 4). During the cold season, PM_10_ measurements exhibited strong agreement with the EM Fidas analyser (R^2^ = 0.74–0.77), whereas substantially weaker relationships were observed during the warm season (R^2^ = 0.40–0.44). In addition, the regression slopes were considerably lower during the warm season, indicating systematic underestimation of PM_10_ concentrations. A similar seasonal pattern was observed for PM_2.5_ measurements; however, the overall agreement with the reference method was substantially stronger, with R^2^ values ranging from 0.85 to 0.94 during the cold season and from 0.72 to 0.82 during the warm season (Figure 5).

The observed seasonal differences were further confirmed by the SPR analysis (Table 3). For PM_10_, median SPR values ranged from 0.81 to 0.99 during the cold season but decreased to 0.36–0.41 during the warm season, indicating substantial underestimation of PM_10_ concentrations under warm-season conditions. Similar but less pronounced behaviour was observed for PM_2.5_, where median SPR values decreased from 1.01 to 1.18 in the cold season to 0.65–0.75 in the warm season. Seasonal differences were highly significant for all sensors (Wilcoxon’s rank-sum test, *p* < 0.001) and were associated with large effect sizes, with Cliff’s delta (δ) values ranging from 0.74 to 0.76 for PM_10_ and from 0.69 to 0.71 for PM_2.5_.

A more detailed analysis demonstrated that seasonality alone explained only part of the observed variability in SPR. Therefore, additional regression models incorporating specific meteorological variables were evaluated (see Table 4). Model M2, which included T, RH and their interaction term, substantially improved the explained variability compared to the season-only model M1 for both PM_10_ and PM_2.5_ measurements. This finding indicates that meteorological conditions contribute considerably to the seasonal variability in sensor response. The influence of temperature and relative humidity on SPR is further illustrated by observed and model-predicted temperature–humidity response surfaces (Figures S7 and S8 in the Supplementary Materials), which show reduced sensor performance (SPR < 0.5) under warm and relatively dry conditions and increased SPR values (SPR > 2) under cold and humid conditions. Among the tested models, M2 generally provided the highest coefficients of determination (R^2^) and the lowest AIC values. Model M3, based on the DPD, achieved very similar explanatory power while requiring fewer explanatory variables (Table 4). Because DPD integrates the combined effects of T and RH into a single physically meaningful parameter and reduces collinearity between meteorological predictors, M3 was finally considered the most suitable model for the interpretation of meteorological influences on sensor performance. Similar results were achieved in the case of PM_2.5_ SPR evaluation (see Table S8 in the Supplementary Materials).

**Figure 6:**
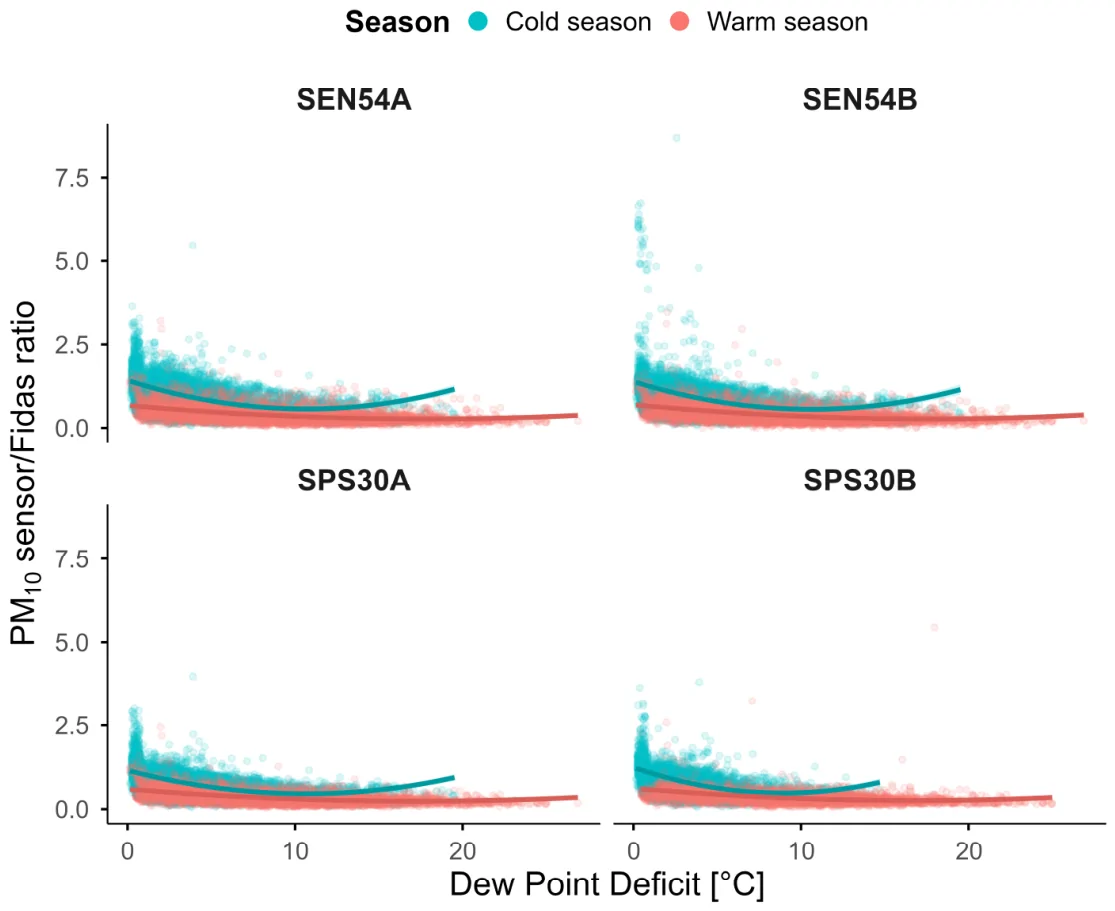
The relationship between the PM_10_ sensor/Fidas ratio (sensor performance ratio) and the dew point deficit (DPD in °C) during the cold season (blue) and the warm season (red). The non-linear relationship results from the quadratic term in the DPD variable are also shown (M3 model in Table 4).

The relationship between PM_10_ SPR and DPD is shown in Figure 6. For all the sensors tested, SPR exhibited a pronounced non-linear dependence on DPD. The SPR tended to be elevated at very low DPD values (DPD ≈ 0, SPR ~ 1–1.5) consistent with enhanced particle hygroscopic growth under humid conditions. A minimum ratio was observed at intermediate DPD values (DPD 5–12 °C), while the SPR increased again under very dry conditions (DPD > 15 °C). A similar but considerable weaker effect was also detected in PM_2.5_ SPR (Figure S9 in the Supplementary Materials).

PM_2.5_ and PM_10_ particle counts reported by the SPS30 sensors showed strong correlations with corresponding EM Fidas PM_2.5_ and PM_10_ particle counts but substantial underestimation throughout the study period (Table S9 in the Supplementary Materials). Coefficients of determination (R^2^) ranged from 0.77 to 0.86, while RMSE values ranged from 280 to 342 particles·m^−3^. Regression slopes were approximately 0.30 for both PM_10_ and PM_2.5_ particle counts (Table S10 in the Supplementary Materials). Both season and DPD remained significant predictors of the particle-count SPR, with effect sizes and model performance comparable to those obtained for PM mass concentrations (Table S11 and Figures S10–S13 in the Supplementary Materials).

### 3.4. Performance of PM Sensor-Correction Models

The performance of the main tested MARS correction models during the two-month training phase (the practical scenario) is summarised in the Supplementary Materials, with PM_10_ results presented in Table S12 and PM_2.5_ results in Table S13. For both PM_10_ and PM_2.5_, CM3 (DPD-based correction) and CM1 (temperature- and relative humidity-based correction) achieved nearly identical performance, with R^2^ values typically around 0.82 and 0.95, respectively, and comparable GCV errors (generally close to 42 for PM_10_ and 8 for PM_2.5_). Inclusion of the interaction between temperature and relative humidity (CM2) did not result in a consistent improvement. Although the model achieved a comparable performance during the training phase (Table S12 in the Supplementary Materials), its performance on the independent testing dataset was generally poorer than that of CM1 and CM3 and deteriorated substantially for SPS30A (Table 5). For the SPS30 sensors, the alternative particle count-based model (MC4) yielded slightly higher R^2^ values and lower GCV errors than the mass concentration-based models while requiring fewer model terms (Table 5 and Table 6).

**Figure 7:**
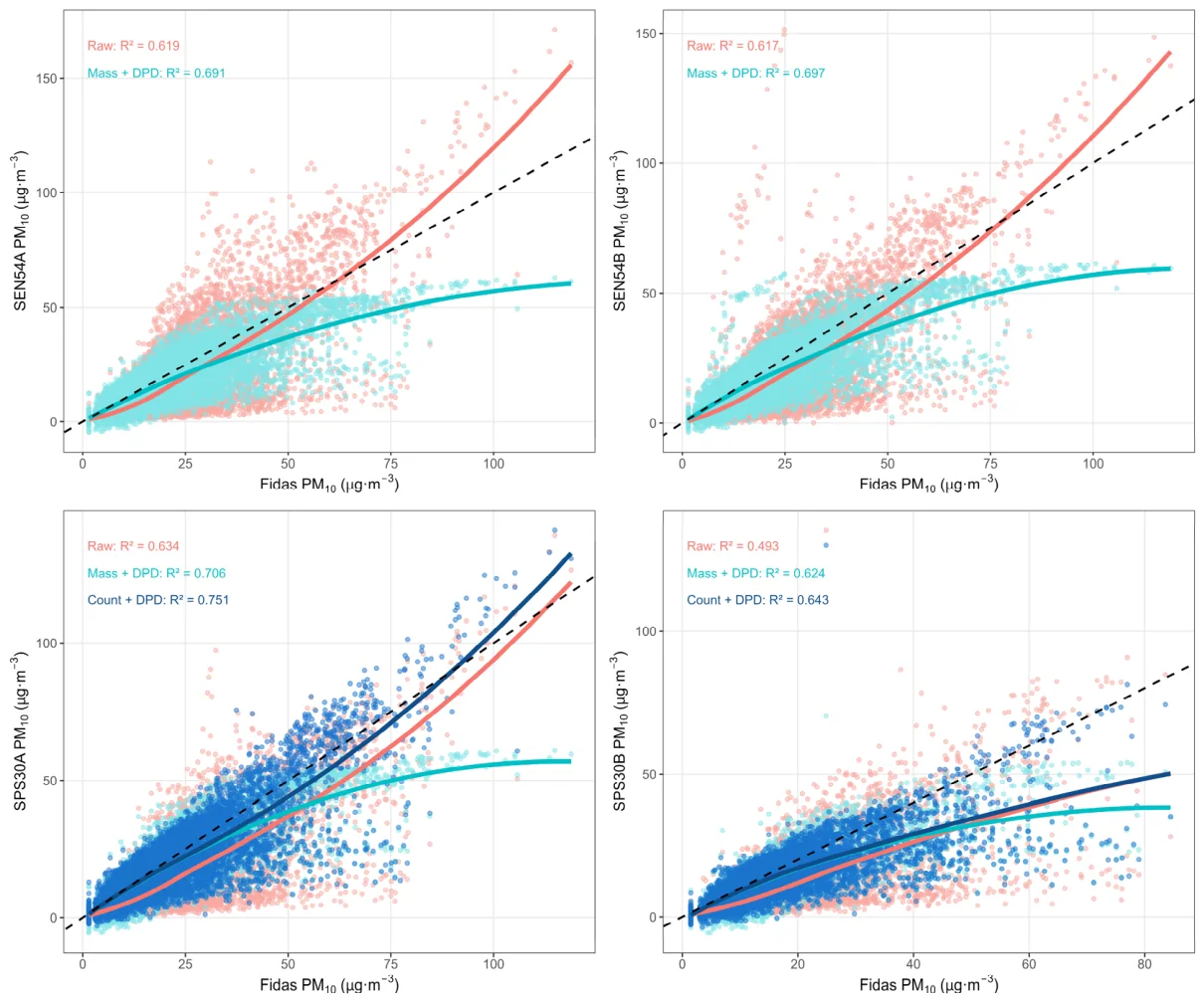
Relationship between PM_10_ concentrations measured by individual sensors (*y*-axis) and the reference Fidas monitor (*x*-axis). Raw sensor measurements (red) are compared with concentrations corrected using sensor-reported PM mass concentration and dew point deficit (Mass + DPD, light blue) and with a particle count-based correction combined with dew point deficit (Count + DPD, dark blue). Correction models were developed using a two-month training dataset (1 February–30 March 2024), whereas model performance was evaluated using an independent testing dataset (31 March 2024–31 August 2025). R^2^ values obtained from linear regression are shown in each panel. The dashed black line represents the 1:1 relationship. LOESS smoothing curves (span = 0.90) are included to illustrate trends in sensor response.

**Figure 8:**
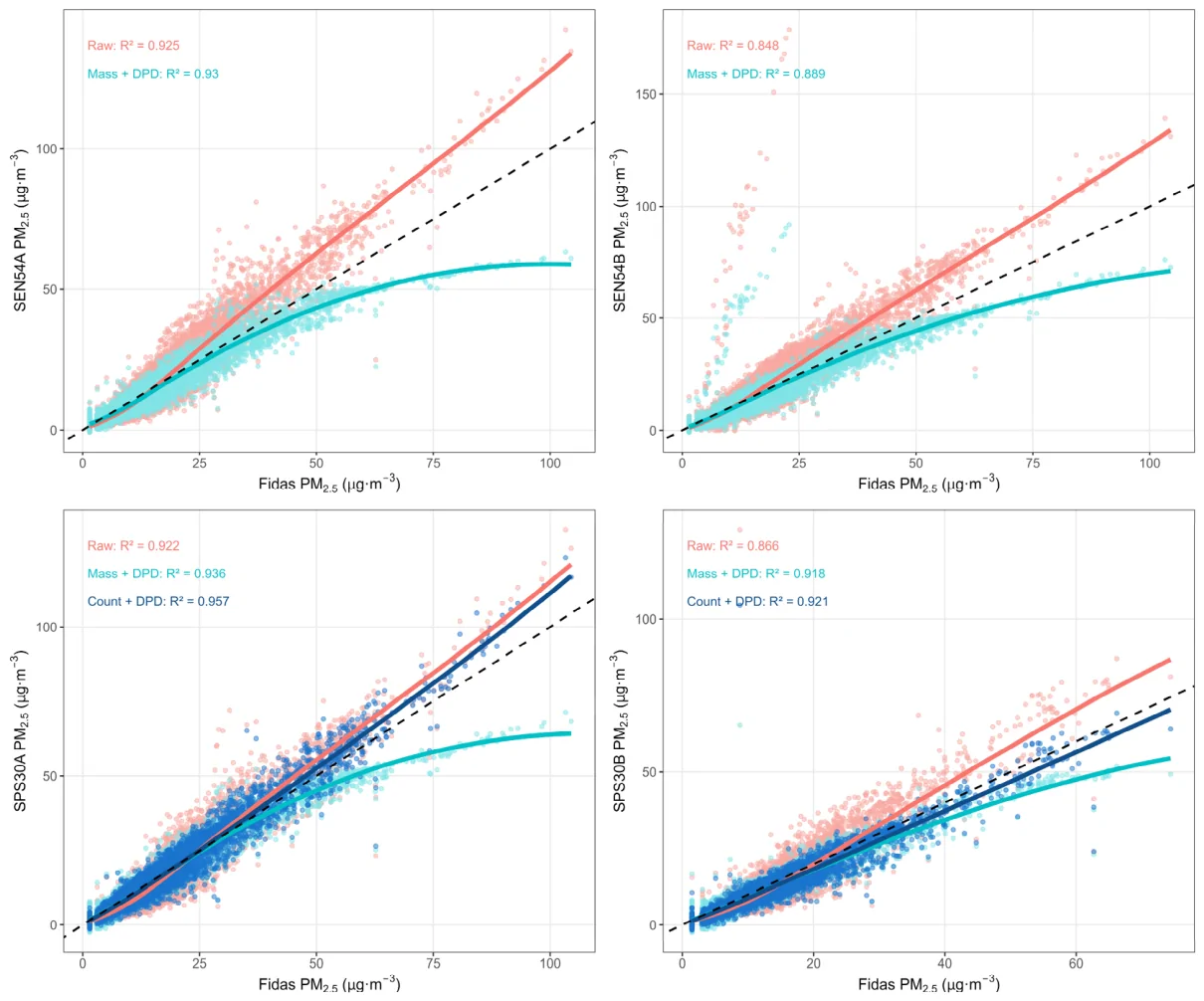
Relationship between PM_2.5_ concentrations measured by individual sensors (*y*-axis) and the equivalent Fidas monitor (*x*-axis). Raw sensor measurements (red) are compared with concentrations corrected using sensor-reported PM mass concentration and dew point deficit (mass + DPD, light blue) and with a particle count-based correction combined with dew point deficit (count + DPD, dark blue). Correction models were developed using a two-month training dataset (1 February–30 March 2024), whereas model performance was evaluated using an independent testing dataset (31 March 2024–31 August 2025). R^2^ values obtained from linear regression are shown in each panel. The dashed black line represents the 1:1 relationship. LOESS smoothing curves (span = 0.90) are included to illustrate trends in sensor response.

For PM_10_, the DPD-based correction (CM3) substantially improved overall agreement with the EM Fidas compared with the raw sensor measurements (CM0). The R^2^ increased from 0.49 to 0.63 to 0.62–0.71, while RMSE decreased from 11.2 to 12.3 to 7.8–8.6 µg·m^−3^. Similarly, MAE decreased from 8.3 to 9.0 to 5.1–5.7 µg·m^−3^. The improvement was observed consistently across both SEN54 and SPS30 sensors, indicating that atmospheric moisture conditions represented by DPD explain a substantial fraction of the variability affecting sensor response (Table 5; Figure 7). For PM_2.5_, the DPD-based correction resulted in a smaller improvement because the raw sensor measurements already showed high agreement with the EM Fidas (R^2^ = 0.85–0.93, RMSE = 4.2–7.1 µg·m^−3^, MAE = 2.9–3.6 µg·m^−3^). After correction, the R^2^ values increased to 0.89–0.94, while RMSE and MAE decreased to 2.7–4.0 µg·m^−3^ and 1.8–2.2 µg·m^−3^, respectively (Table 6, Figure 8).

**Figure 9:**
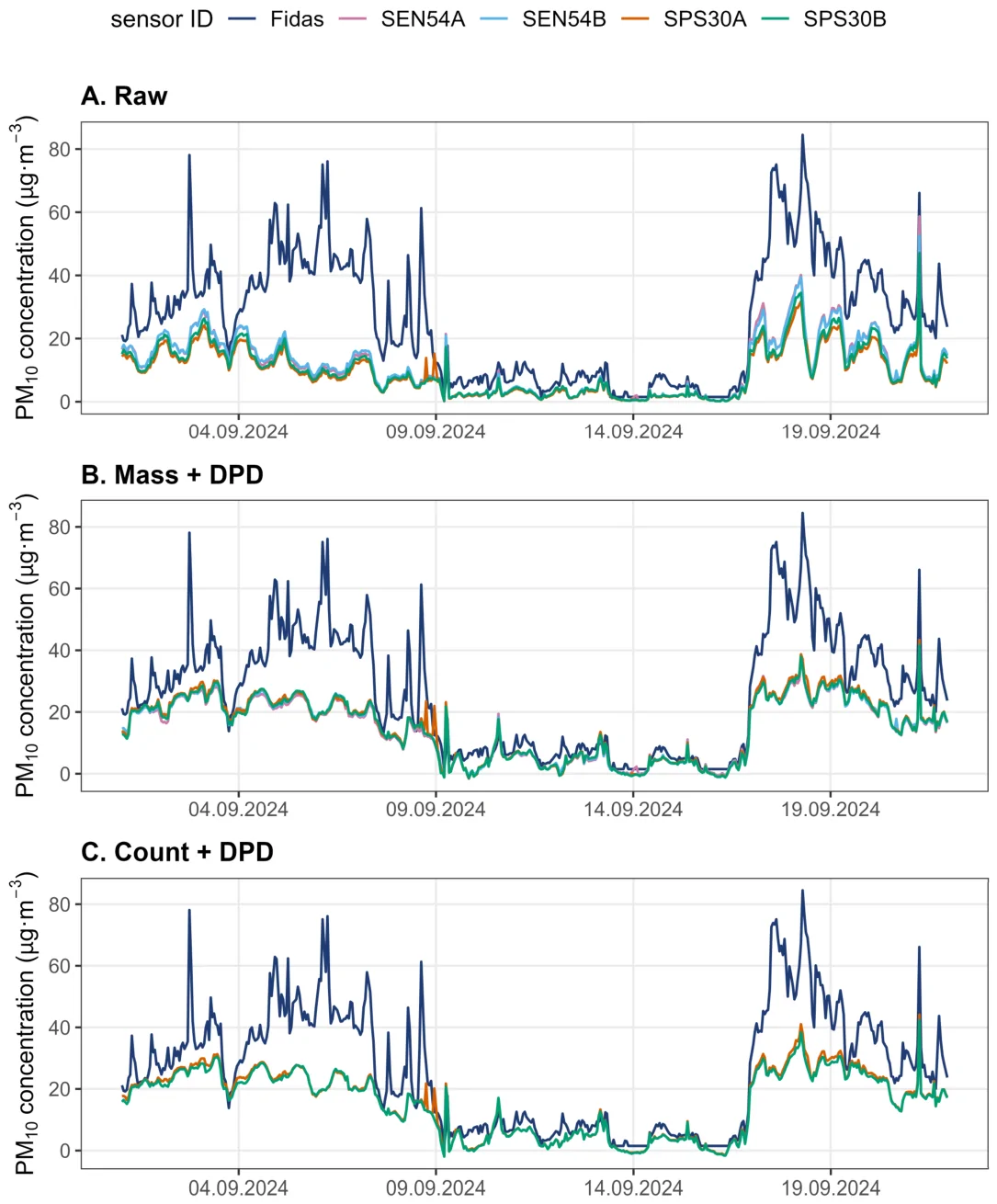
Representative time series of PM_10_ concentrations measured by different sensors and the reference Fidas measurement during a warm-season episode (1–21 September 2024). Panel A shows raw sensor measurements, panel B shows concentrations corrected using sensor-reported PM mass concentration and dew point deficit (Mass + DPD), and panel C shows concentrations corrected using particle-count variables combined with the dew point deficit (Count + DPD). Corrected concentrations were obtained using MARS models developed from a two-month training dataset (1 February–30 March 2024).

**Figure 10:**
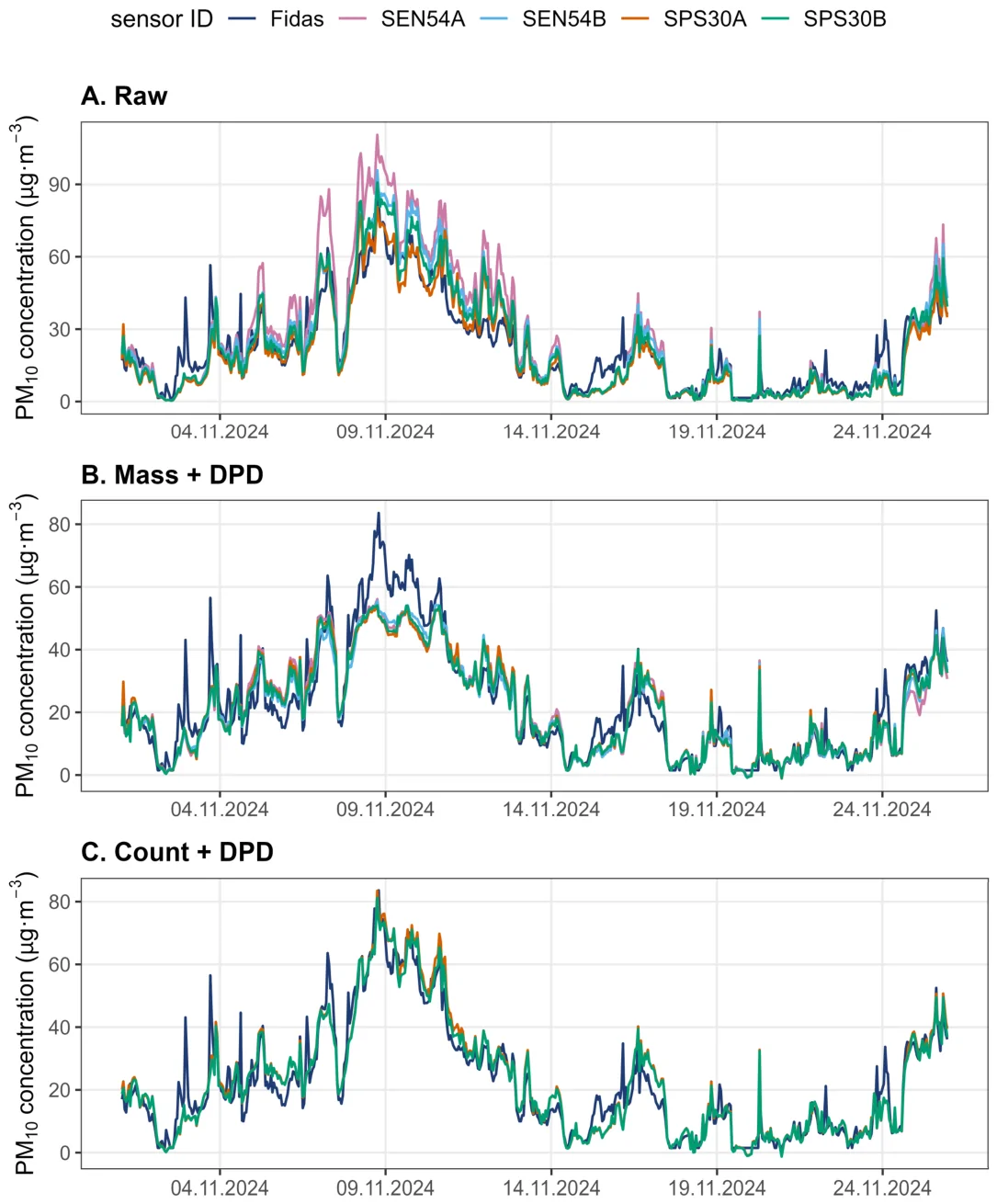
Representative time series of PM_10_ concentrations measured by different sensors and the reference Fidas measurement during a cold-season episode (1–25 November 2024). Panel A shows raw sensor measurements, panel B shows concentrations corrected using sensor-reported PM mass concentration and dew point deficit (Mass + DPD), and panel C shows concentrations corrected using particle-count variables combined with the dew point deficit (Count + DPD). Corrected concentrations were obtained using MARS models developed from a two-month training dataset (1 February–30 March 2024).

The visual inspection of the regression plots revealed that the DPD-based correction model tended to increasingly underestimate higher PM concentrations. To further investigate the robustness of this model, the dataset was divided into observations with PM concentrations ≤50 and >50 µg·m^−3^ according to the EM Fidas measurements. The threshold was selected as an exploratory diagnostic criterion corresponding to the concentration range where corrected values began to systematically deviate from the 1:1 relationship (Figure 7 and Figure 8). For PM_10_, the DPD-based correction substantially improved sensor performance at concentrations ≤ 50 µg·m^−3^. Across all sensors, R^2^ increased from 0.45 to 0.50 to 0.61–0.65, while the RMSE decreased from 10.2 to 11.1 to 6.7–7.1 µg·m^−3^ and the MAE from 7.8 to 8.3 to 4.7–5.1 µg·m^−3^ (n = 7910–10,954; Table 7). However, at concentrations > 50 µg·m^−3^ (n = 168–536), the correction consistently underestimated the reference measurements, resulting in increased negative bias (e.g., from −13.05 to −17.74 µg·m^−3^ for SPS30A), regression slopes decreasing from values close to unity to 0.32 or less, and coefficients of determination approaching zero (see Table 7 and Figure 9 and Figure 10).

A comparable—although less pronounced—pattern was observed for PM_2.5_. At concentrations ≤ 50 µg·m^−3^ (n = 8024–11,241), the DPD-based correction increased R^2^ from 0.78 to 0.89 to 0.86–0.93 and reduced the RMSE from 4.0 to 6.7 to 2.5–3.7. Regression slopes also improved from 1.10 to 1.26 to 0.89–0.97, indicating closer agreement with the EM Fidas measurement (Table 8; Figures S14 and S15 in the Supplementary Materials). However, the performance of the DPD-based correction deteriorated at PM_2.5_ concentrations > 50 µg·m^−3^ (n = 53–245). In several cases, regression statistics were poorer than those obtained for the raw sensor data. For SPS30A, for example, the regression slope decreased from approximately 1.26 to 0.45, while the intercept increased from −10.70 to 22.96 (Table 8; Figures S14 and S15 in the Supplementary Materials).

Alternative MARS models including the season and hour of day were also tested using an extended-training (6-month) scenario (1 February–31 July 2024). Under these conditions, both season and hour provided further slight improvements in model performance, particularly when combined with DPD (see Table S14 for PM_10_ and Table S15 for PM_2.5_ in the Supplementary Materials). The best results in the case of PM_10_ were obtained for the model incorporating DPD, season and hour of the day, including pairwise interactions (resulting in R^2^ 0.65–0.70, RMSE 6.86–7.38, slope 0.66–0.70; Table S16 and Figure S16 in the Supplementary Materials). In the case of PM_2.5_, the best results were obtained for the model incorporating DPD, season and hour (R^2^ 0.90–0.93, RMSE 2.50–4.16, slope 0.84–0.89; Table S17 and Figure S17 in the Supplementary Materials). In both scenarios (for the two-month or six-month training dataset), T and RH correction models yielded a very similar performance to the DPD-based correction models (for example, SEN54a in Figure S18 in the Supplementary Materials), including cases where the corrections failed for concentrations higher than 50 µg·m^−3^ (see the complete results for PM_10_ and PM_2.5_ in Tables S18 and S19 in the Supplementary Materials, respectively). Additional particle count- and DPD-based models for SPS30 sensors resulted in further improvements in overall PM_10_ model performance. Implementation of sensor PM_1_ counts increased the R^2^ from 0.62 to 0.71 to 0.64–0.75, reduced the RMSE from 7.79 to 8.16 to 7.52–7.65 µg·m^−3^ and increased the regression slope from 0.63–0.71 to 0.68–0.89 (Table 5; Figure 7). Similar improvements were observed for PM_2.5_, resulting in R^2^ values of 0.92–0.96, RMSE values of around 2.6 µg·m^−3^, slopes close to one, and intercepts close to zero (Table 6; Figure 8). Evaluation within low- and high-concentration ranges indicated that the additional particle-count information was particularly beneficial for SPS30A PM_10_ measurements at elevated concentrations (>50 µg·m^−3^), whereas improvements for SPS30B in this concentration range remained very limited and high-concentration PM_10_ events continued to be poorly reproduced (Table 7). The performance of the sensors within low- and high-concentration ranges is summarised in Table 7 and Table 8 and Figure 7 and Figure 8 (complete results are provided in Tables S18 and S19 in the Supplementary Materials). Examples of time series of PM_10_ concentrations during a warm-season episode (September 2024) are shown in Figure 9. Raw sensor data substantially underestimated the EM Fidas measurements, particularly during periods of elevated concentrations. Application of the sensor mass concentration + DPD correction improved agreement with the reference instrument and considerably reduced differences between individual sensors. The sensor count + DPD approach provided additional improvements, especially during concentration peaks, although residual underestimation remained. In contrast, an example of cold-season conditions (November 2024; Figure 10) showed considerably better agreement between the sensor and EM Fidas measurements, both before and after correction. Corrected sensor concentrations closely followed the temporal variability of the EM Fidas across a broad concentration range, with substantially smaller residual deviations than those observed during the September episode. Similar patterns, although with a smaller magnitude of bias, as in the case of PM_10_, were observed in the case of PM_2.5_ measurement (Figures S14 and S15 in the Supplementary Materials).

## 4. Discussion

### 4.1. Sensirion PM Sensors Performance and Seasonality Effect

The results of this study showed substantially weaker performances of both Sensirion sensor types for PM_10_ than for PM_2.5_ measurements. During several episodes characterised by exceptionally high concentrations of coarse particles, identified by the reference Fidas monitor as Saharan dust intrusions or resuspension events associated with nearby open-cast coal mining activities, neither the SPS30 nor the SEN54 sensors adequately captured the observed PM_10_ peaks. The identification of these episodes was supported by complementary analyses using ceilometer observations, satellite imagery and web camera records. Similarly to previous studies [3,8,17,35], these events were excluded from further performance evaluation, as the observed discrepancies reflected a known limitation of optical particle counters rather than ordinary measurement uncertainty. Because these coarse-particle episodes were not adequately represented in the sensor signal, they could not be reliably addressed by the correction procedures applied in this study. Nevertheless, even after excluding these episodes, PM_10_ measurements remained considerably less accurate (mean R^2^ = 0.60, mean RMSE = 10.0 µg·m^−3^) than PM_2.5_ measurements (mean R^2^ = 0.90, mean RMSE = 4.6 µg·m^−3^). This difference is expected because PM_10_ concentrations were not measured directly by the tested sensors but were derived from measurements of smaller particle fractions and thus were more strongly affected by coarse-particle populations, which represent a known challenge for low-cost optical particle counters. These findings are consistent with previous studies reporting a substantially better performance of SPS30 sensors for the fine particle fractions PM_1_ and PM_2.5_ than for PM_10_ [13,14,15,16]. Several studies have also identified SPS30 as one of the most stable commercially available low-cost OPCs, exhibiting low inter-sensor variability and generally better reproducibility than many Plantower, Honeywell and Sharp sensor types [12,13,14]. Despite this generally favourable performance, pronounced seasonal variability was observed in the present study, with substantially better agreement between sensor and EM Fidas measurements during the cold season (October–March) and systematic underestimation during the warm season (April–September). Seasonal differences were further assessed using the sensor performance ratios (SPRs) calculated separately for the two seasons. The observed deterioration in performance was substantial, particularly for PM_10_, where median SPR values decreased from 0.81–0.99 during the cold season to 0.36–0.41 during the warm season, with all sensors exhibiting large effect sizes (Cliff’s δ = 0.74–0.76). Similar warm-season underestimation was reported for Plantower PMS7003 sensors by [8], who attributed this behaviour to changes in particle size distribution, particle density and other aerosol properties affecting the conversion of optical measurements into PM mass concentrations. Likewise, refs. [36,37] highlighted the importance of seasonal variability in the evaluation of low-cost PM sensor performance.

The pronounced seasonal variability observed in both SPS30 and SEN54 measurements suggests that meteorological conditions play a major role in determining sensor performance. In agreement with numerous previous studies, air temperature and relative humidity were identified as the most important environmental variables influencing the agreement between sensor and reference measurements [7,13,38]. The influence of atmospheric humidity is commonly attributed to hygroscopic growth of aerosol particles, which alters particle size, optical properties and, consequently, the light-scattering signal detected by optical particle counters [4,18]. However, the relationship between sensor performance and atmospheric humidity is often non-linear and became increasingly pronounced only above certain humidity thresholds [7,39]. This is not surprising because hygroscopic growth itself is inherently non-linear and depends on both the hygroscopic properties of aerosol particles and the proximity of air to saturation [18].

### 4.2. Dew Point Deficit as an Explanatory Variable

In the present study, the dew point deficit (DPD) integrates temperature and humidity into a single physically interpretable variable describing both proximity to water saturation and overall atmospheric moisture conditions. The seasonal separation observed in the DPD distributions confirms that atmospheric moisture conditions differed markedly between the warm and cold seasons. For all sensors, the relationship between SPR and DPD was clearly non-linear. The largest deviations from unity in the sensor/reference ratio were generally observed at low DPD values, corresponding to conditions close to saturation, where hygroscopic particle growth is expected to have the greatest influence on optical measurements. However, the non-linear relationship between sensor/reference ratio and DPD was weaker than would be expected for an uncorrected optical sensor response under humid conditions. This observation is consistent with previous studies and suggests that modern Sensirion sensors may already incorporate internal processing algorithms that partially compensate for humidity-related measurement artefacts [13,17,40]. At the same time, a clear separation between warm- and cold-season observations remained visible across much of the DPD range. The persistence of reduced SPR values during the warm season, even at moderate DPD levels (5–12 °C), indicates that atmospheric moisture alone cannot fully explain the observed seasonal variability in sensor performance. Nevertheless, the strong overall relationship between sensor performance and DPD suggests that atmospheric moisture remains one of the key factors influencing sensor response.

The SPS30 sensors further enable the analysis of particle counts for PM_10_ and PM_2.5_ aerosol fractions. Interestingly, the SPS30 particle counts were substantially lower than those measured by the EM Fidas monitor throughout the study period. It should be noted that particle counts reported by the SPS30 and EM Fidas are not strictly equivalent measurements, as they are derived using different particle detection and sizing approaches. The median SPS30 counts corresponded to only about 17% of the EM Fidas counts, while the mean values reached approximately 22% of those reported by Fidas (for both PM_10_ and PM_2.5_ counts). Despite this systematic underestimation, strong correlations between SPS30 and EM Fidas particle counts were observed (R^2^ = 0.77–0.86), indicating that the sensors were able to reproduce temporal variability well. These results suggest that discrepancies between the two instruments arise not only from the conversion of particle counts into mass concentrations but also from limitations associated with particle detection and sizing. Similar limitations have been reported previously for low-cost optical particle counters. Ref. [18] highlighted the importance of particle size distribution information for accurately representing aerosol behaviour under changing humidity conditions, while [14] concluded that low-cost optical sensors exhibit restricted particle-sizing capabilities, particularly for larger particles. Therefore, particle-count measurements should not be interpreted as direct estimates of ambient particle abundance, but rather as surrogate variables that retain valuable information on aerosol variability and can provide useful predictors for sensor correction models.

### 4.3. Effect of Dew Point Defict on MARS Correction Model Performance

Building on the findings of a previous study [1], this study evaluated the applicability of DPD as a correction variable for PM sensor data using MARS models. DPD-based correction achieved a performance comparable to models using temperature and relative humidity as separate predictors (Table 4 and Table 5; Figure S18 in the Supplementary Materials). Therefore, DPD was retained as the preferred correction variable due to its simpler physical interpretability and the elimination of collinearity between multiple explanatory meteorological predictors. The benefits of DPD-based correction were particularly evident for PM_10_, where the CM3 models increased R^2^ from 0.49–0.63 for raw measurements to 0.62–0.71 and reduced RMSE from 11.2–12.3 to 7.79–8.60 µg·m^−3^. Improvements for PM_2.5_ were less pronounced, reflecting the already good quality of the raw sensor measurements. Overall, the results indicate that DPD can serve as an effective single-variable alternative to the conventional combination of temperature and relative humidity in PM sensor correction models.

Although overall error statistics still improved, the correction models increasingly underestimated elevated PM events, particularly for PM_10_. This behaviour was evident in the regression plots, where corrected concentrations increasingly deviated from the ideal 1:1 relationship at higher PM levels because the fitted correction functions tended to level off (see Figure 7 and Figure 8).

For both PM_10_ and PM_2.5_, DPD-based correction substantially improved agreement with the EM Fidas monitor for concentrations ≤ 50 µg·m^−3^, reflected by higher R^2^ values and lower RMSE and MAE. In contrast, concentrations above 50 µg·m^−3^ remained more challenging. Although overall error statistics still improved, the correction models increasingly underestimated elevated PM events, particularly for PM_10_.

A likely explanation is the limited representation of high-concentration observations in both the training and the validation datasets. Measurements exceeding 50 µg·m^−3^ typically accounted for less than 5% of observations, depending on the sensor type and PM fraction. Consequently, model behaviour in this concentration range should be interpreted with caution. Nevertheless, the tendency toward increasing underestimation at elevated PM concentrations was observed consistently across all the tested sensors. Similar limitations of statistical correction approaches have been reported by [41], who observed reduced performance of humidity-based correction models during extreme PM_2.5_ episodes.

The inclusion of additional predictors such as the season and hour of the day provided little additional benefit. Even in the extended-training scenario (6 months), these variables did not substantially improve model performance and failed to resolve the persistent underestimation of high PM_10_ and PM_2.5_ concentrations (Tables S14 and S15 and Figures S16 and S17 in the Supplementary Materials).

### 4.4. Particle Counts Versus PM Mass Concentrations

Following the approaches proposed by [21,23], particle-count variables were incorporated together with DPD into MARS correction models and compared with models based solely on sensor-reported PM mass concentrations. The inclusion of particle-count information provided additional improvements for both PM_10_ and PM_2.5_ measurements of the SPS30 sensors. For PM_10_, the R^2^ values increased from 0.62 to 0.71 for the DPD-based models to 0.64–0.75 when particle-count variables were added, while the RMSE decreased from 7.79 to 8.16 to 7.52–7.65 µg·m^−3^. The largest improvement was observed for SPS30A, where R^2^ increased from 0.71 to 0.75 and the regression slope shifted from 0.71 to 0.89, indicating substantially improved reproduction of elevated PM_10_ concentrations (see Figure 7). The additional benefit of particle-count information became particularly apparent during elevated PM episodes (>50 µg·m^−3^), although the magnitude of improvement differed between sensors. While SPS30A showed a more accurate reproduction of concentration peaks, improvements for SPS30B remained limited (Table 7; Figure 7).

Similar—although generally smaller—improvements were observed for PM_2.5_. The incorporation of particle-count data increased the R^2^ from 0.92 to 0.94 to 0.92–0.96 and reduced the RMSE from 2.67 to 3.39 to 2.61–2.70 µg·m^−3^. In addition, the regression slopes moved closer to the ideal value of 1, particularly for SPS30A, where the slope increased from 0.90 to 1.05. The additional benefit of particle-count information became particularly apparent during elevated PM episodes (>50 µg·m^−3^), where mass-based corrections continued to underestimate concentrations, whereas count-based models reproduced the concentration peaks more accurately (see Figure 8).

Although the SPS30 particle counts alone substantially underestimated the EM Fidas measurements, their contribution to the correction models demonstrates that they retain information relevant for PM estimation that is not fully preserved in sensor-reported PM mass concentrations. This interpretation is consistent with [21,23], who suggested that information may be lost during the internal conversion of particle counts into PM mass concentrations. Similarly, ref. [18] emphasised the importance of particle-size-distribution information and demonstrated that humidity-related corrections are physically more robust when applied to particle size distributions rather than PM mass concentrations. Together, these findings suggest that particle-count information provides an additional source of information on aerosol characteristics beyond that contained in sensor-reported PM mass concentrations.

### 4.5. Remaining Residual Bias of Sensors

Despite the improvements achieved by both DPD-based and particle count-based correction models, residual underestimation remained evident, particularly during the warm season (Figure 9; Figure S14 in the Supplementary Materials), whereas substantially better agreement with the EM Fidas monitor was observed during the cold season (Figure 10; Figure S15 in the Supplementary Materials). This pattern persisted even after the incorporation of particle-count information, indicating that neither atmospheric moisture conditions nor particle-count information alone are sufficient to fully explain the observed variability in sensor response. Previous studies have shown that the relationship between optical measurements and PM mass concentration depends strongly on aerosol properties, including particle size distribution, refractive index, density and chemical composition [24,25]. In addition, hygroscopic growth processes themselves depend on aerosol composition and may therefore vary substantially between seasons [18]. The persistence of systematic warm-season underestimation observed in the present study may therefore reflect seasonal changes in aerosol characteristics that were not captured by the predictor variables used.

Furthermore, the limited improvement achieved by introducing additional predictors such as season and hour of day suggests that these variables may act only as indirect proxies for underlying aerosol processes. Similar limitations have been reported for low-cost optical particle counters during periods dominated by atypical aerosol populations and coarse particles [8], highlighting the continuing challenges associated with the development of universally applicable correction models for optical PM sensors.

## 5. Conclusions

Significant seasonal differences were observed in the response of both PM_10_ and PM_2.5_ optical sensors. A substantial proportion of this variability could be explained by atmospheric moisture conditions represented by the dew point deficit (DPD), which provided a simple, physically interpretable alternative to conventional correction approaches based on temperature and relative humidity. The relationship between the DPD and sensor response was non-linear, supporting the application of MARS-based correction models. DPD-based correction substantially improved PM_10_ measurements and also improved PM_2.5_ measurements under typical ambient conditions, although the benefits were less pronounced because of the already strong performance of the raw PM_2.5_ measurements. However, both PM fractions remained underestimated during some high-concentration pollution episodes (>50 µg·m^−3^). The results indicate that, despite the improvements achieved by the correction models, low-cost optical particle counters remain less reliable for monitoring PM_10_ than PM_2.5_, particularly under conditions dominated by coarse particles and elevated PM concentrations. For SPS30 sensors, particle-count information provided additional improvements in PM concentration estimates, particularly for PM_10_ measurements, although high PM_10_ episodes remained challenging for some sensors. Overall, the results demonstrate that DPD is an effective explanatory variable for optical PM sensor correction and that particle-count information provides additional value for improving PM concentration estimates, particularly under elevated pollution conditions. Residual warm-season underestimation indicates that factors beyond atmospheric moisture, likely related to aerosol properties, continue to influence sensor response.

## Figures and Tables

**Table 1:** Description of different MARS models tested for correction of PM_10_ and PM_2.5_ sensor measurements. T = temperature; RH = relative humidity; DPD = dew point deficit. CM4 models were used only for the SPS30 sensor types.

Model ID	Variable	Correction Model Description
CM0	PM_10_	PM_10_ Fidas ~ PM_10_ sensor
CM1		PM_10_ Fidas ~ PM_10_ sensor + T + RH
CM2		PM_10_ Fidas ~ PM_10_ sensor + T * RH
CM3		PM_10_ Fidas ~ PM_10_ sensor + DPD
CM4		PM_10_ Fidas ~ sensor PM_1_ count + DPD
CM0	PM_2.5_	PM_2.5_ Fidas ~ PM_2.5_ sensor
CM1		PM_2.5_ Fidas ~ PM_2.5_ sensor + T + RH
CM2		PM_2.5_ Fidas ~ PM_2.5_ sensor + T * RH
CM3		PM_2.5_ Fidas ~ PM_2.5_ sensor + DPD
CM4		PM_2.5_ Fidas ~ sensor PM_2.5_ count + DPD

**Table 2:** Performance statistics for raw PM_10_ and PM_2.5_ concentrations measured by SEN54 and SPS30 sensors compared with the equivalent monitor Palas Fidas 200S during the entire study period (1 February 2024 to 31 August 2025). Performance metrics include the coefficient of determination (R^2^), root mean square error (RMSE), mean absolute error (MAE), bias, and the slope and intercept of linear regression.

Sensor	n	R^2^	RMSE	MAE	Bias	Slope	Intercept
SEN54A PM_10_	12,701	0.52	14.15	9.21	−4.37	0.84	−1.19
SEN54B PM_10_	12,673	0.52	13.58	8.53	−4.84	0.78	−0.40
SPS30A PM_10_	12,649	0.54	13.51	8.89	−7.30	0.66	−0.63
SPS30B PM_10_	9283	0.42	13.55	8.57	−6.85	0.56	0.96
SEN54A PM_2.5_	12,653	0.91	5.61	3.56	0.41	1.24	−2.78
SEN54B PM_2.5_	12,673	0.84	7.18	3.70	0.91	1.24	−2.27
SPS30A PM_2.5_	12,649	0.91	4.47	3.06	−1.18	1.09	−2.37
SPS30B PM_2.5_	9283	0.85	4.85	3.09	−0.73	1.14	−2.34

**Table 3:** Seasonal variations in the medians of the sensor performance ratio (SPR) for raw PM_10_ and PM_2.5_ measurements obtained from all the sensors tested. Difference = SPR cold − SPR warm season, Clif. Delta = Cliff’s delta effect size measure. Effect size = a verbal description of effect size. Seasonal differences were assessed using the Wilcoxon rank-sum test and were found to be statistically significant in all cases (*p* < 0.001).

Variable	Sensor	SPR Median Cold Season	SPR Median Warm Season	Difference	Cliff. Delta	Effect Size
PM_10_	SEN54A	0.99	0.40	0.59	0.76	large
	SEN54B	0.98	0.41	0.57	0.76	large
	SPS30A	0.81	0.36	0.45	0.76	large
	SPS30B	0.83	0.38	0.45	0.74	large
PM_2.5_	SEN54A	1.15	0.72	0.43	0.71	large
	SEN54B	1.18	0.75	0.43	0.69	large
	SPS30A	1.01	0.65	0.36	0.71	large
	SPS30B	1.09	0.68	0.41	0.70	large

**Table 4:** Results of three main models tested for sensor performance in the case of PM_10_ measurement. SPR = sensor performance ratio, season (cold or warm), RH = relative humidity (%), T = temperature (°C), DPDc = mean-centred dew point deficit (°C). Model results: R^2^ = coefficient of determination; AIC = Akaike Information Criterion; Adj. R^2^ = adjusted coefficient of determination.

Model ID	Model Description	Sensor ID	Model Results		
			R^2^	AIC	Adj. R^2^
M1	PM_10_ SPR ~ season	SEN54A	0.40	9908.5	0.40
		SEN54B	0.32	12,569.1	0.32
		SPS30A	0.39	3297.9	0.39
		SPS30B	0.37	3396.5	0.37
M2	PM_10_ SPR ~ season + RH * T	SEN54A	0.57	5597.1	0.57
		SEN54B	0.47	9427.2	0.47
		SPS30A	0.60	−2070.6	0.60
		SPS30B	0.58	−429.3	0.58
M3	PM_10_ SPR ~ season + DPDc + DPDc^2^	SEN54A	0.52	7021.7	0.52
		SEN54B	0.44	10,257.9	0.44
		SPS30A	0.54	−280.7	0.54
		SPS30B	0.52	893.0	0.52

**Table 5:** Performance of PM_10_ correction models on an independent testing dataset (practical scenario: from 31 March 2024 to 31 August 2025). Results are shown for the tested correction models and individual sensors, including the number of valid observations (n), coefficient of determination (R^2^), root mean square error (RMSE), mean absolute error (MAE), bias, and the slope and intercept of the linear regression between corrected sensor concentrations and EM Fidas measurements.

Model ID	Model Predictors	Sensor	n	R^2^	RMSE	MAE	Bias	Slope	Intercept
CM0	PM_10_ sensor	SEN54A	11,490	0.62	12.30	8.96	−4.43	1.02	−4.88
		SEN54B	11,438	0.62	11.78	8.35	−4.81	0.95	−3.85
		SPS30A	11,486	0.63	11.60	8.64	−7.12	0.80	−3.32
		SPS30B	8078	0.49	11.15	8.30	−6.91	0.68	−1.61
CM1	PM_10_ sensor + T + RH	SEN54A	11,490	0.70	7.90	5.38	−1.13	0.67	5.09
		SEN54B	11,438	0.71	7.76	5.22	−0.41	0.69	5.59
		SPS30A	11,486	0.71	7.67	5.39	−0.11	0.70	5.67
		SPS30B	8078	0.63	7.04	4.81	−0.47	0.64	5.53
CM2	PM_10_ sensor + T * RH	SEN54A	11,490	0.47	11.67	8.81	3.73	0.64	10.64
		SEN54B	11,438	0.51	11.06	8.19	3.64	0.65	10.23
		SPS30A	11,486	0.00	120.20	71.49	67.53	0.32	80.51
		SPS30B	8078	0.36	11.45	8.84	4.55	0.62	10.84
CM3	PM_10_ sensor + DPD	SEN54A	11,490	0.69	8.60	5.72	−3.28	0.69	2.69
		SEN54B	11,438	0.70	8.43	5.46	−2.98	0.70	2.76
		SPS30A	11,486	0.71	8.16	5.38	−2.58	0.71	2.89
		SPS30B	8078	0.62	7.79	5.07	−3.32	0.63	2.88
CM4	sensor PM_1_ count + DPD	SPS30A	11,486	0.75	7.65	5.08	−1.63	0.89	0.53
		SPS30B	8078	0.64	7.52	4.76	−3.01	0.68	2.37

**Table 6:** Performance of PM_2.5_ correction models on an independent testing dataset (practical scenario: from 31 March 2024 to 31 August 2025). Results are shown for the tested correction models and individual sensors, including the number of valid observations (n), the coefficient of determination (R^2^), the root mean square error (RMSE), the mean absolute error (MAE), bias, and the slope and intercept of the linear regression between corrected sensor concentrations and EM Fidas measurements.

Model ID	Model Predictors	Sensor	n	R^2^	RMSE	MAE	Bias	Slope	Intercept
CM0	PM_2.5_ sensor	SEN54A	11,442	0.93	5.23	3.41	0.27	1.26	−3.09
		SEN54B	11,438	0.85	7.08	3.57	0.81	1.27	−2.60
		SPS30A	11,486	0.92	4.18	3.00	−1.17	1.11	−2.62
		SPS30B	8078	0.87	4.21	2.90	−0.97	1.17	−2.69
CM1	PM_2.5_ sensor + T + RH	SEN54A	11,442	0.93	3.41	2.19	−1.25	0.86	0.48
		SEN54B	11,438	0.88	4.16	2.23	−0.77	0.89	0.60
		SPS30A	11,486	0.94	3.07	1.99	−0.81	0.90	0.49
		SPS30B	8078	0.92	2.75	1.89	−1.29	0.87	0.09
CM2	PM_2.5_ sensor + T * RH	SEN54A	11,442	0.94	3.12	1.95	−0.23	0.84	1.74
		SEN54B	11,438	0.89	3.89	2.06	0.26	0.86	2.03
		SPS30A	11,486	0.94	3.05	2.08	0.51	0.87	2.16
		SPS30B	8078	0.92	2.51	1.77	0.39	0.85	1.91
CM3	PM_2.5_ sensor + DPD	SEN54A	11,442	0.93	3.39	2.17	−1.10	0.86	0.67
		SEN54B	11,438	0.89	4.02	2.13	−0.99	0.89	0.47
		SPS30A	11,486	0.94	3.06	1.95	−0.62	0.90	0.71
		SPS30B	8078	0.92	2.67	1.81	−1.08	0.87	0.27
CM4	sensor PM_2.5_ count + DPD	SPS30A	11,486	0.96	2.70	1.83	−0.27	1.05	−0.97
		SPS30B	8078	0.92	2.61	1.71	−1.11	0.94	−0.46

**Table 7:** Performance of PM_10_ measurements in two concentration ranges (≤50 and >50 μg·m^−3^) before and after correction. Raw indicates original sensor data, cor indicates correction based on sensor-reported PM mass concentration and dew point deficit (DPD), and cor2 indicates correction based on particle-count variables and DPD (both developed using a two-month training dataset). Performance statistics were calculated using an independent testing dataset (31 March 2024–31 August 2025). Statistical metrics (R^2^, RMSE, MAE, bias, regression slope, and intercept) were calculated relative to the reference Fidas monitor; n = number of valid observations.

Sensor	Concentration Ranges	n	R^2^	RMSE	MAE	Bias	Slope	Intercept
SEN54A PM_10_	raw ≤ 50	10,954	0.48	11.10	8.32	−4.67	0.93	−3.47
	cor ≤ 50	10,954	0.62	7.14	5.07	−2.52	0.75	1.73
	raw > 50	536	0.24	26.90	21.93	0.38	1.26	−16.32
	cor > 50	536	0.11	23.30	18.95	−18.90	0.32	23.76
SEN54B PM_10_	raw ≤ 50	10,902	0.47	10.81	7.81	−4.89	0.86	−2.47
	cor ≤ 50	10,902	0.62	7.04	4.86	−2.26	0.75	1.91
	raw > 50	536	0.24	24.11	19.01	−3.16	1.15	−12.42
	cor > 50	536	0.08	22.58	17.64	−17.59	0.29	26.94
SPS30A PM_10_	raw ≤ 50	10,950	0.50	10.61	8.20	−6.83	0.73	−2.26
	cor ≤ 50	10,950	0.65	6.74	4.77	−1.84	0.79	1.65
	cor2 ≤ 50	10,950	0.67	6.52	4.61	−1.49	0.83	1.33
	raw > 50	536	0.24	24.18	17.45	−13.05	0.98	−11.93
	cor > 50	536	0.08	22.39	17.77	−17.74	0.27	28.14
	cor2 > 50	536	0.29	19.62	14.63	−4.48	1.03	−6.37
SPS30B PM_10_	raw ≤ 50	7910	0.45	10.20	7.92	−6.64	0.67	−1.52
	cor ≤ 50	7910	0.61	6.69	4.66	−2.87	0.68	2.08
	cor2 ≤ 50	7910	0.63	6.47	4.41	−2.66	0.70	2.01
	raw > 50	168	0.01	32.80	26.40	−19.60	0.23	27.20
	cor > 50	168	0.00	28.45	24.37	−24.36	0.03	34.33
	cor2 > 50	168	0.01	27.34	21.17	−19.14	0.24	27.26

**Table 8:** Performance of PM_2.5_ measurements in two concentration ranges (≤50 and >50 μg·m^−3^) before and after correction. Raw indicates original sensor data, cor indicates correction based on sensor-reported PM mass concentration and dew point deficit (DPD), and cor2 indicates correction based on particle-count variables and DPD. Performance statistics were calculated using an independent testing dataset (31 March 2024–31 August 2025). Statistical metrics (R^2^, RMSE, MAE, bias, regression slope, and intercept) were calculated relative to the reference Fidas monitor; n = number of valid observations.

Sensor	Concentration Ranges	n	R^2^	RMSE	MAE	Bias	Slope	Intercept
SEN54A PM_2.5_	raw ≤ 50	11,197	0.89	4.71	3.17	−0.01	1.26	−3.00
	cor ≤ 50	11,197	0.92	2.70	1.95	−0.87	0.93	−0.02
	raw > 50	245	0.77	16.32	14.46	13.34	1.38	−9.68
	cor > 50	245	0.61	14.28	11.90	−11.89	0.35	27.45
SEN54B PM_2.5_	raw ≤ 50	11,193	0.78	6.73	3.32	0.52	1.26	−2.53
	cor ≤ 50	11,193	0.86	3.67	1.96	−0.79	0.94	−0.11
	raw > 50	245	0.79	16.48	14.77	13.99	1.36	−7.61
	cor > 50	245	0.80	11.75	10.22	−10.21	0.56	16.49
SPS30A PM_2.5_	raw ≤ 50	11,241	0.89	3.98	2.90	−1.30	1.10	−2.48
	cor ≤ 50	11,241	0.93	2.48	1.76	−0.40	0.97	−0.05
	cor2 ≤ 50	11,241	0.94	2.50	1.74	−0.35	1.04	−0.82
	raw > 50	245	0.76	9.70	7.81	4.96	1.26	−10.70
	cor > 50	245	0.75	12.52	10.52	−10.50	0.45	22.96
	cor2 > 50	245	0.81	7.43	5.80	3.14	1.19	−8.16
SPS30B PM_2.5_	raw ≤ 50	8025	0.84	4.05	2.84	−1.05	1.15	−2.52
	cor ≤ 50	8024	0.91	2.49	1.75	−1.02	0.89	0.07
	cor2 ≤ 50	8025	0.91	2.54	1.69	−1.10	0.93	−0.41
	raw > 50	53	0.14	14.88	12.97	10.42	0.87	17.74
	cor > 50	53	0.03	12.19	10.68	−10.68	0.16	37.30
	cor2 > 50	53	0.17	7.84	4.93	−2.79	0.66	16.37

## Data Availability

The data presented in this paper are not publicly available because they were acquired using sensor devices borrowed from a third party and are not owned by the authors’ institution. The datasets are available from the corresponding author upon reasonable request.

## Supplementary Materials

The following supporting information can be downloaded at https://www.mdpi.com/article/10.3390/s26216914/s1.
