# Supplementary material for: Long-Term Field Performance of Sensirion SEN54 and SPS30 Low-Cost Particle Sensors: Seasonal Variability and Correction Using Dew Point Deficit
Source: Sensors (Basel). 2026 Oct 31;26(21):6914. doi: 10.3390/s26216914 (PMC13766482; doi:10.3390/s26216914)
Supplement: Supplementary file 1 [file sensors-26-06914-s001.zip › sensors-4547728-supplementary.pdf]

Supplementary Materials for

Long-Term Field Performance of Sensirion SEN54 and SPS30 Low-Cost Particle Sensors: Seasonal Variability and Correction Using Dew Point Deficit

Petra Tesařová *, Adriana Šindelářová, Josef Keder, Petr Vincenc, Štěpán Rychlík

Czech Hydrometeorological Institute, Na Šabatce 2050/17, 143 06 Prague 4, Czech Republic

* Correspondence: petra.tesarova@chmi.cz

Table S1. Descriptive statistics of temperature, relative humidity and dew point deficit during the cold season (October–March) and warm season (April–September) at the Tušimice measurement site during the Sensirion sensor testing campaign (1 February 2024 to 31 August 2025).

Season	Variable	N	Mean ± SD	Median	Min	Max
Cold	Dew Point Deficit (°C)	5768	3.46 ± 2.97	2.78	0.14	19.54
	Relative Humidity (%)	5768	79.84 ± 14.3	82.17	23.67	99.00
	Temperature (°C)	5768	4.30 ± 5.08	4.07	-14.10	18.78
Warm	Dew Point Deficit (°C)	8051	7.16 ± 4.94	6.00	0.15	26.91
	Relative Humidity (%)	8051	65.75 ± 18.48	67.33	19.17	99.00
	Temperature (°C)	8051	16.55 ± 6.22	16.42	-4.37	35.22

Table S2. Description of alternative MARS correction models tested for PM_{10} and $PM_{2.5}$ sensor measurements under the extended-training scenario. DPD = dew point deficit, season = cold or warm season, hour = hour of the day, interaction = pairwise (second-order) interactions among all model terms. Models CM0e–CM5e were developed and evaluated using a 6 month training dataset (1 February–31 July 2024).

Model ID	Variable	Correction model description
CM0e	PM_{10}	PM_{10} Fidas ~ PM_{10} sensor
CM1e		PM_{10} Fidas ~ PM_{10} sensor + DPD
CM2e		PM_{10} Fidas ~ PM_{10} sensor + DPD + season
CM3e		PM_{10} Fidas ~ PM_{10} sensor + DPD + hour
CM4e		PM_{10} Fidas ~ PM_{10} sensor + DPD + season + hour
CM5e		PM_{10} Fidas ~ PM_{10} sensor + DPD + season + hour + interaction
CM0e	$PM_{2.5}$	$PM_{2.5}$ Fidas ~ $PM_{2.5}$ sensor
CM1e		$PM_{2.5}$ Fidas ~ $PM_{2.5}$ sensor + DPD
CM2e		$PM_{2.5}$ Fidas ~ $PM_{2.5}$ sensor + DPD + season
CM3e		$PM_{2.5}$ Fidas ~ $PM_{2.5}$ sensor + DPD + hour
CM4e		$PM_{2.5}$ Fidas ~ $PM_{2.5}$ sensor + DPD + season + hour
CM5e		$PM_{2.5}$ Fidas ~ $PM_{2.5}$ sensor + DPD + season + hour + interaction

Table S3. Descriptive statistics of hourly PM_{10} and $PM_{2.5}$ concentrations (in $\mu g \cdot m^{-3}$) measured by individual Sensirion sensors and by the monitor equivalent-to-reference Fidas throughout the entire testing period (1 February 2024–31 August 2025). n = number of valid samples, Q_1–Q_3 = first and third quartiles, Max = maximum hourly concentration.

Sensor ID	n	Mean	Median	Q_1–Q_3	Max
SEN54A PM_{10}	12747	15.34	7.81	3.76–18.66	171.23
SEN54B PM_{10}	12719	14.86	8.04	3.85–18.77	151.47
SPS30A PM_{10}	12695	12.44	6.75	3.45–15.11	139.49
SPS30B PM_{10}	9309	10.91	6.28	3.32–13.04	152.27
**Fidas PM_{10}**	**13733**	**19.27**	**15.10**	**9.30–24.20**	**261.50**
SEN54A $PM_{2.5}$	12699	13.53	7.50	3.66–17.13	142.41
SEN54B $PM_{2.5}$	12719	14.02	7.89	3.80–17.89	178.94
SPS30A $PM_{2.5}$	12695	11.94	6.69	3.41–14.82	132.43
SPS30B $PM_{2.5}$	9309	10.54	6.24	3.30–12.90	129.07
**Fidas $PM_{2.5}$**	**13733**	**12.77**	**9.20**	**5.30–16.00**	**104.50**

Table S4. Correlation matrix of PM_{10} concentrations measured by sensors (SEN54A, SEN54B, SPS30A and SPS30B) and by equivalent monitor Palas Fidas 200S throughout the entire testing period (1 February 2024–31 August 2025). Spearman correlation coefficients (r_S) are shown.

Variable	PM_{10} Fidas	PM_{10} SEN54A	PM_{10} SEN54B	PM_{10} SPS30A
PM_{10} SEN54A	0.64			
PM_{10} SEN54B	0.66	0.99		
PM_{10} SPS30A	0.66	0.99	0.98	
PM_{10} SPS30B	0.65	0.98	0.98	0.99

Table S5. Correlation matrix of $PM_{2.5}$ concentrations measured by sensors (SEN54A, SEN54B, SPS30A and SPS30B) and by equivalent monitor Palas Fidas 200S throughout the entire testing period (1 February 2024–31 August 2025). Spearman correlation coefficients (r_S) are shown.

Variable	$PM_{2.5}$ Fidas	$PM_{2.5}$ SEN54A	$PM_{2.5}$ SEN54B	$PM_{2.5}$ SPS30A
$PM_{2.5}$ SEN54A	0.93			
$PM_{2.5}$ SEN54B	0.93	0.99		
$PM_{2.5}$ SPS30A	0.93	1.00	0.99	
$PM_{2.5}$ SPS30B	0.92	0.99	0.98	0.99

Table S6. Significant episodes with high PM_{10} concentration peaks identified in Tušimice based on EM Fidas measurements throughout the study period (1 February 2024–31 August 2025). The events in March 2024 were characterised by the passage of Saharan dust, which descended to the ground during these periods; other episodes were caused by resuspended coarse particles transported from nearby surface mine.

Date and time of peak (UTC)	Peak duration (h)	Maximum hourly PM_{10} concentration (EM Fidas; $\mu g \cdot m^{-3}$)	Maximum hourly PM_{10} concentration (Sensirion sensors; $\mu g \cdot m^{-3}$)

27.03.2024 18:00	1	86.2	12.4
30.03.2024 11:00	44	205.9	50.9
30.04.2024 21:00	1	86.1	7.7
08.09.2024 16:00	1	126.6	10.2
29.03.2025 14:00	2	261.5	53.5
03.07.2025 9:00	1	99.5	11.3
13.08.2025 8:00	1	89.5	8.4
13.08.2025 18:00	1	83.9	12.8

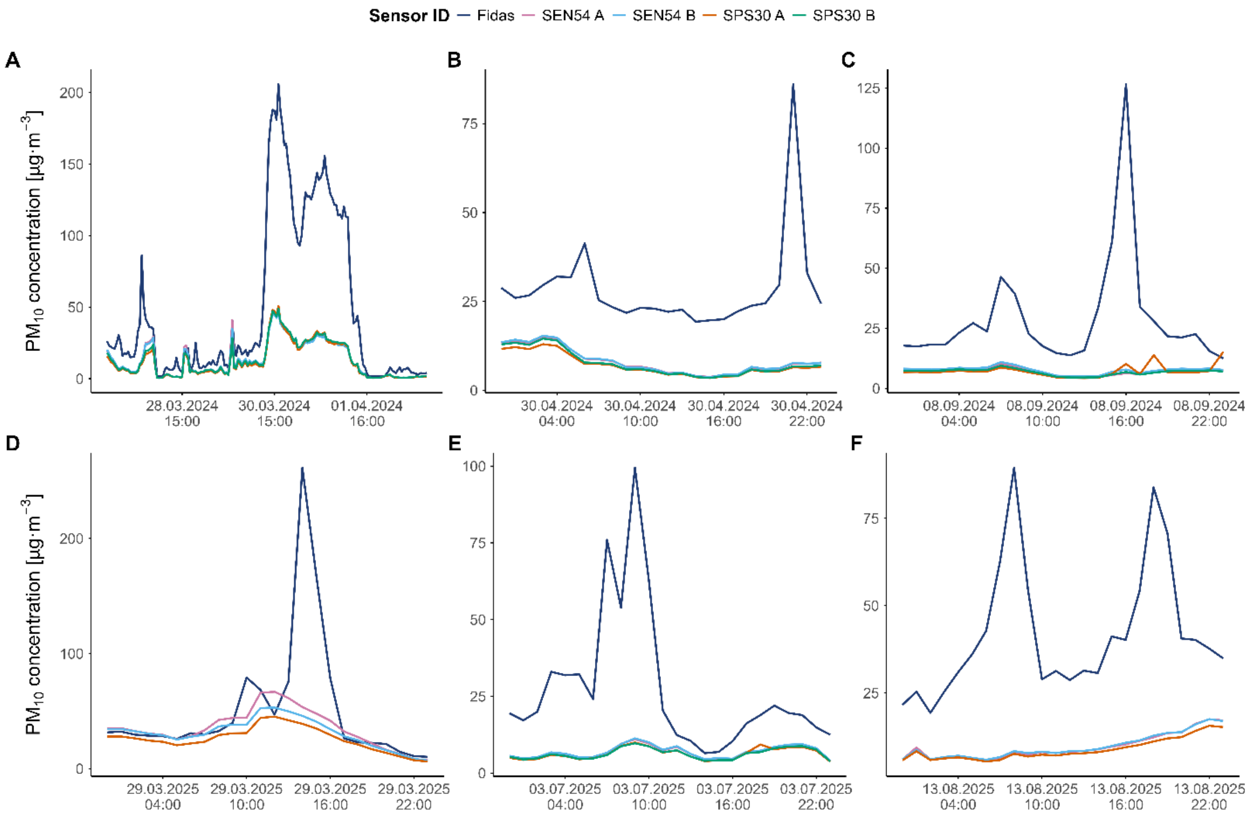

Figure S1. Selected episodes with elevated PM_{10} concentrations recorded by the EM Fidas but not detected by the Sensirion sensors. These episodes were excluded from subsequent analyses of sensor performance across seasons and meteorological conditions (temperature, relative humidity, and dew point deficit), as well as from the development and evaluation of the corresponding correction models.

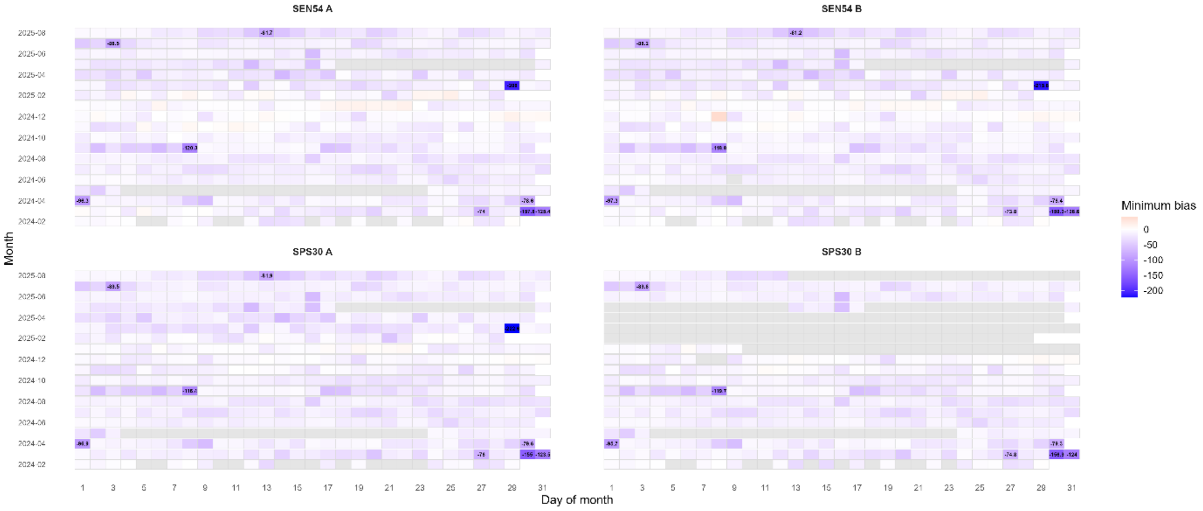

Figure S2. Heatmap showing daily minimum biases in hourly PM_{10} concentrations measured by the individual Sensirion sensors relative to the equivalent-to-reference monitor EM Fidas throughout the entire testing period (1 February 2024–31 August 2025) at the Tušimice monitoring station.

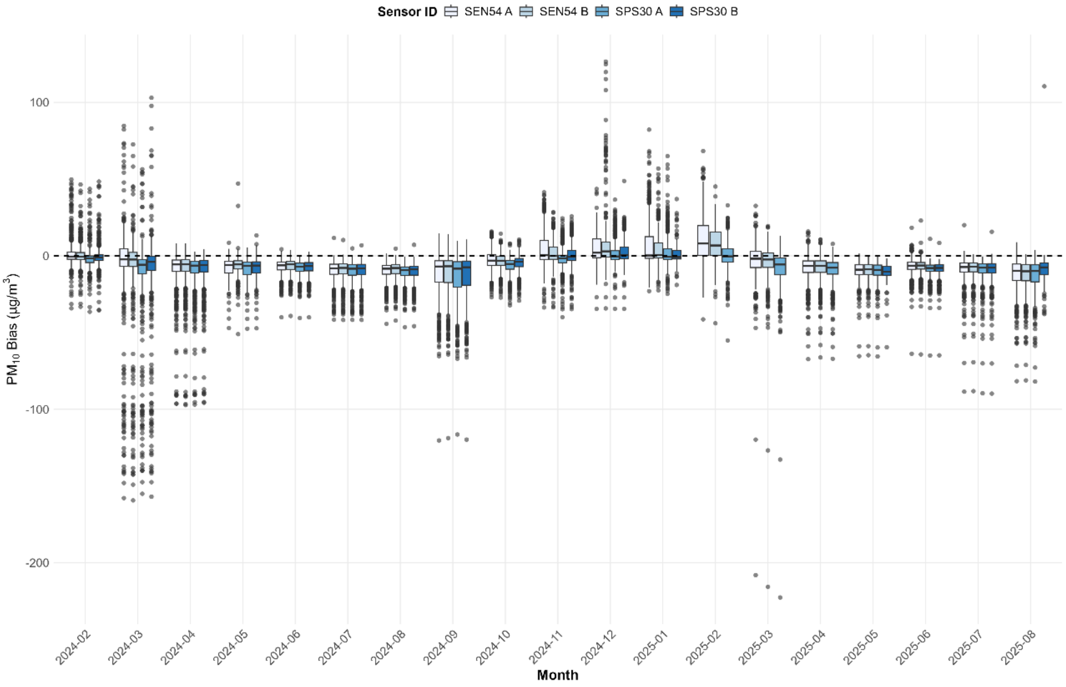

Figure S3. Monthly distribution of hourly PM_{10} measurement bias for individual Sensirion sensors relative to the equivalent-to-reference monitor EM Fidas throughout the entire testing period (1 February 2024–31 August 2025) at the Tušimice monitoring station.

Table S7. Performance statistics for raw PM_{10} and $PM_{2.5}$ concentrations measured by SEN54 and SPS30 sensors compared with the equivalent monitor Palas Fidas 200S after exclusion of Saharan dust and resuspension episodes, corresponding to 52 hourly observations. Reported statistics include the

number of paired observations (n), coefficient of determination (R^2), root mean square error (RMSE), mean absolute error (MAE), bias, and the slope and intercept of the linear regression.

Sensor	n	R^2	RMSE	MAE	Bias	Slope	Intercept
SEN54A PM_{10}	12649	0.63	12.30	8.81	-3.95	1.05	-4.83
SEN54B PM_{10}	12621	0.63	11.58	8.12	-4.41	0.97	-3.74
SPS30A PM_{10}	12597	0.64	11.52	8.48	-6.88	0.81	-3.29
SPS30B PM_{10}	9235	0.53	11.15	8.07	-6.33	0.77	-2.31
SEN54A $PM_{2.5}$	12601	0.92	5.40	3.48	0.51	1.27	-3.06
SEN54B $PM_{2.5}$	12621	0.86	7.02	3.62	1.02	1.27	-2.55
SPS30A $PM_{2.5}$	12597	0.92	4.21	2.97	-1.08	1.12	-2.59
SPS30B $PM_{2.5}$	9235	0.88	4.56	2.99	-0.61	1.20	-2.85

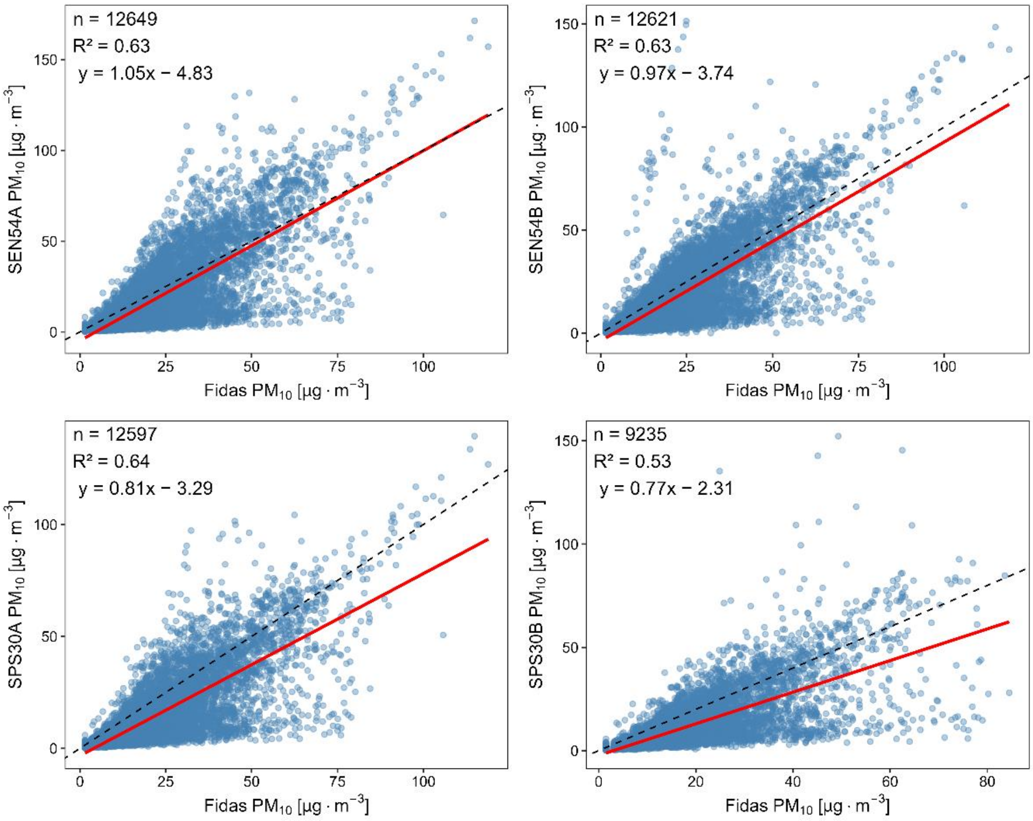

Figure S4. Scatterplots showing the relationship between raw PM_{10} concentrations measured by the four tested Sensirion sensors (SEN54A, SEN54B, SPS30A and SPS30B) and the equivalent-method (EM) Fidas analyser after exclusion of Saharan dust and resuspension episodes. The solid red line represents the linear regression fit, while the dashed black line indicates the 1:1 relationship. Values of n, coefficient of determination (R^2), and regression equation are shown in each panel.

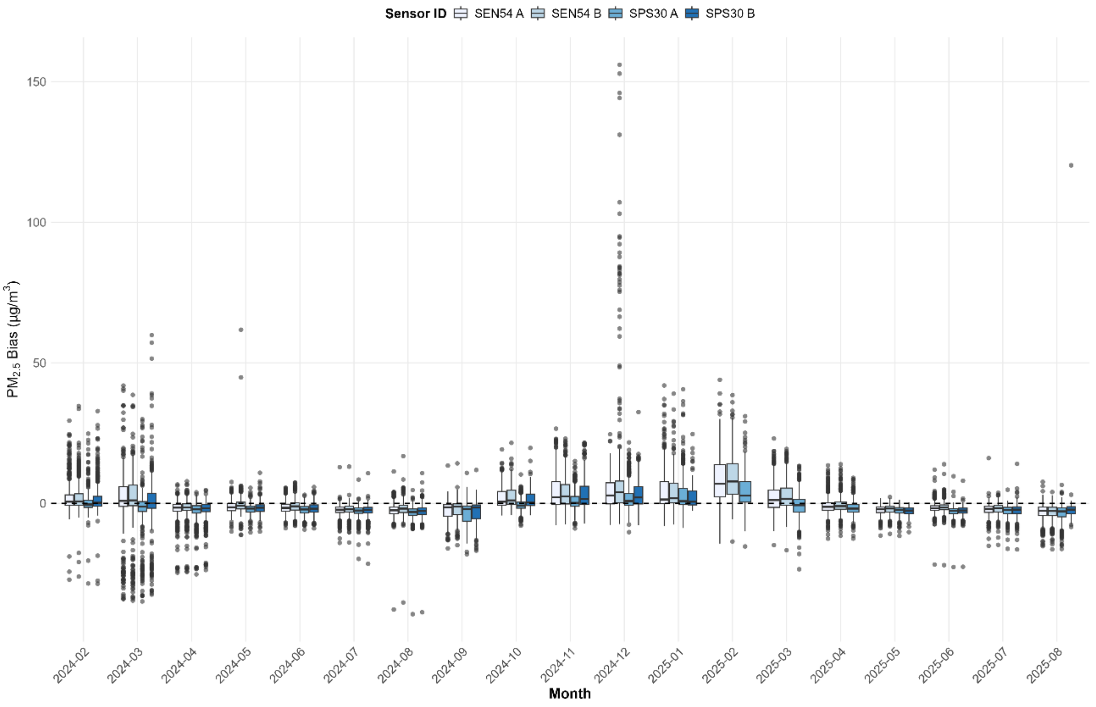

Figure S5. Monthly distribution of hourly $PM_{2.5}$ measurement bias for individual Sensirion sensors relative to the equivalent-to-reference monitor EM Fidas throughout the entire testing period (1 February 2024–31 August 2025) at the Tušimice monitoring station.

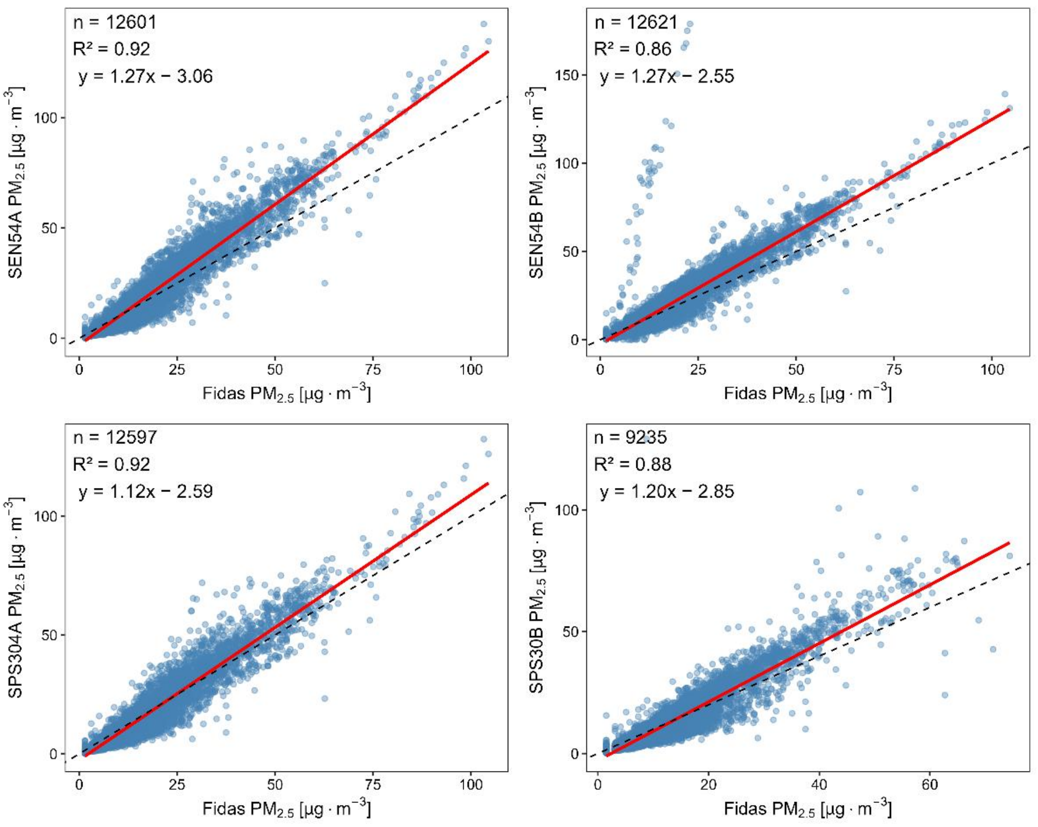

Figure S6. Scatterplots showing the relationship between raw $PM_{2.5}$ concentrations measured by the four tested Sensirion sensors (SEN54A, SEN54B, SPS30A and SPS30B) and the equivalent-method (EM) Fidas analyser after exclusion of Saharan dust and resuspension episodes. The solid red line represents the linear regression fit, while the dashed black line indicates the 1:1 relationship. Values of n, coefficient of determination (R^2), and regression equation are shown in each panel.

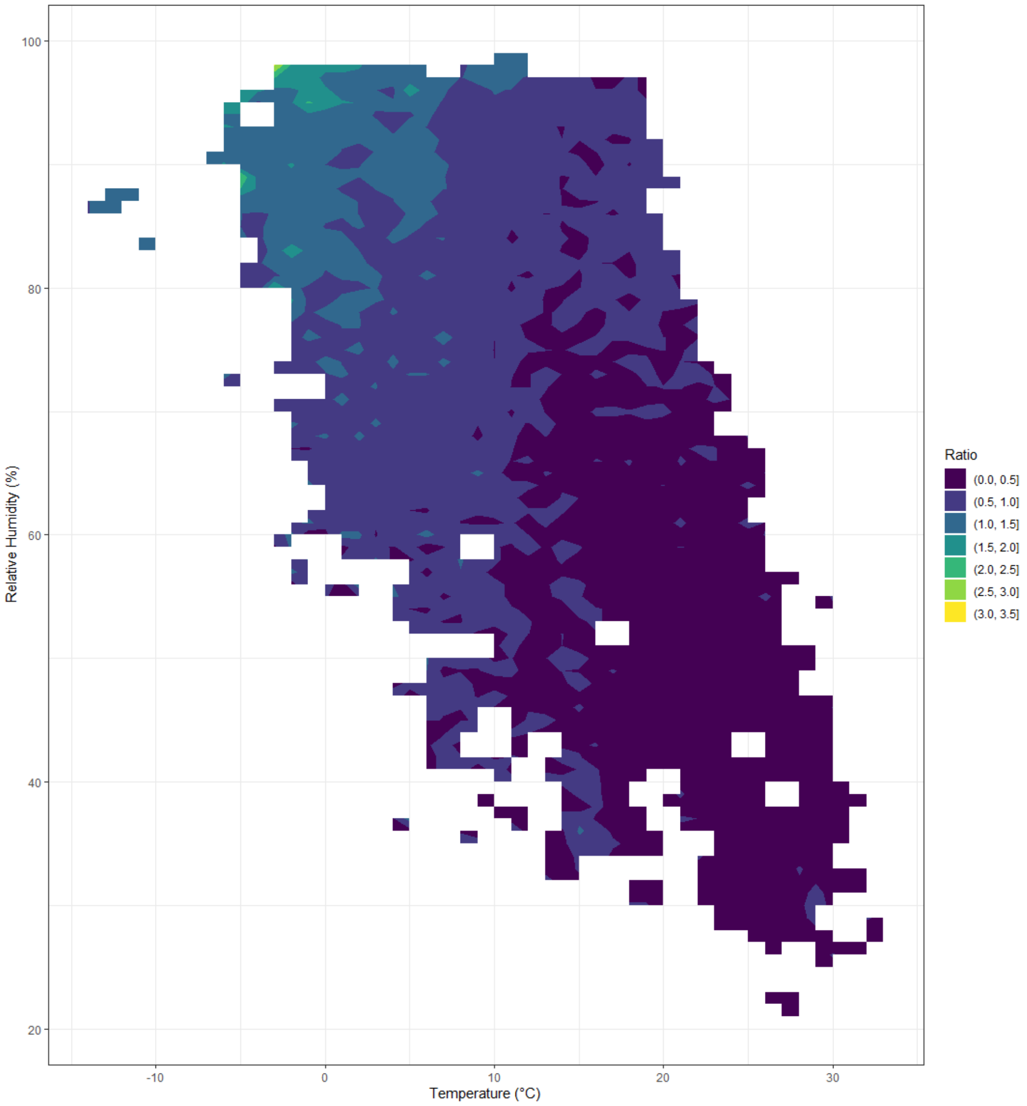

Figure S7. Distribution of observed PM_{10} Sensor Performance Ratio (SPR) values as a function of air temperature and relative humidity during the entire study period. Colours indicate average SPR values within individual temperature-humidity bins.

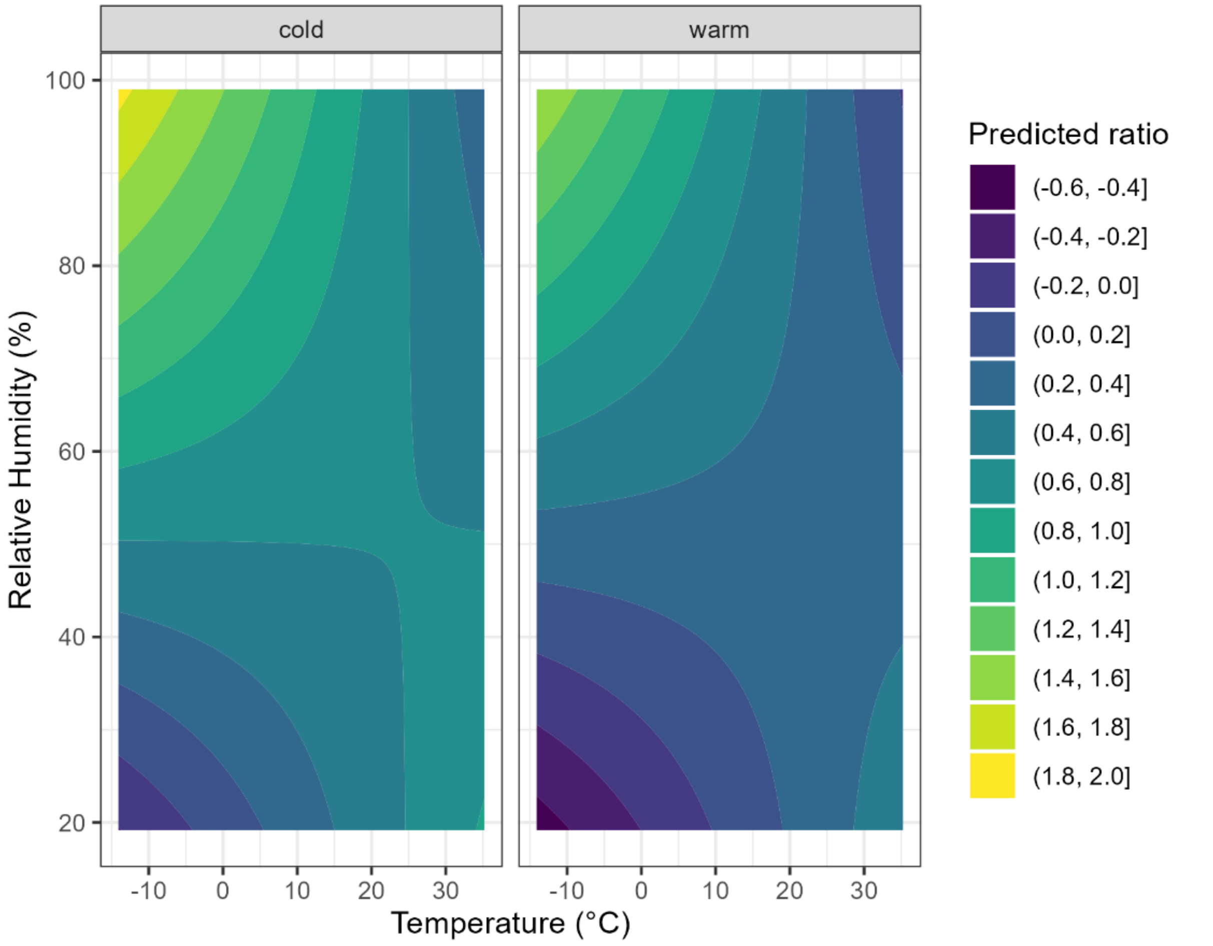

Figure S8. Distribution of predicted SPR values derived from Model M2 (SPR ~ season + RH * T). The contour plots illustrate the combined effects of air temperature and relative humidity on PM_{10} sensor performance in the cold and warm seasons.

Table S8. Results of main three models tested for sensor performance in case of $PM_{2.5}$ measurement. SPR = Sensor Performance Ratio, season (cold or warm), RH = relative humidity (%), T = temperature (°C), DPDc = mean-centred Dew Point Deficit (°C). Model results: R^2 = coefficient of determination, AIC = Akaike Information Criterion, Adj. R^2 = adjusted coefficient of determination.

**Model ID**	**Model despcription**	**Sensor ID**	**Model results**		
			**R^2**	**AIC**	**Adj. R^2**
M4	$PM_{2.5}$ SPR ~ season	SEN54A	0.32	3865.3	0.32
		SEN54B	0.18	15819.5	0.18
		SPS30A	0.31	89.0	0.31
		SPS30B	0.24	5189.1	0.24
M5	$PM_{2.5}$ SPR ~ season + RH * T	SEN54A	**0.50**	184.8	0.50
		SEN54B	**0.29**	13991.3	0.29
		SPS30A	**0.53**	-4746.0	0.53
		SPS30B	**0.41**	2844.1	0.41
M6	$PM_{2.5}$ SPR ~ season + DPDc + DPDc^2	SEN54A	**0.48**	459.1	0.48
		SEN54B	**0.28**	14086.2	0.28
		SPS30A	**0.51**	-4088.9	0.51
		SPS30B	**0.40**	3072.1	0.40

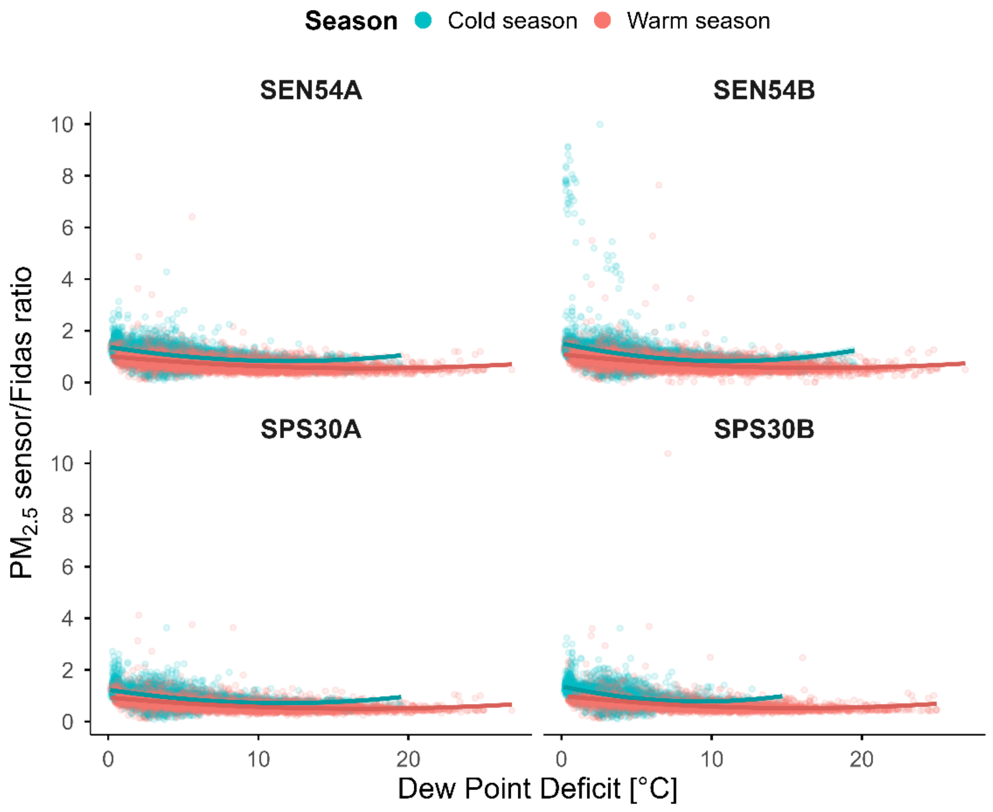

Figure S9. The relationship between the $PM_{2.5}$ Sensor/Fidas ratio (sensor performance ratio) and the dew point deficit (DPD in °C) during the cold season (blue) and the warm season (red). The nonlinear relationship results from the quadratic term in the DPD variable (M6 model in Table S8).

Table S9. Descriptive statistics of hourly PM_{10} and $PM_{2.5}$ particle counts (in particles·m^{-3}) measured by particular Sensirion sensors and by equivalent-to-reference monitor EM Fidas throughout the entire testing period (1 February 2024–31 August 2025). n = number of valid observations, Q_1–Q_3 = first and third quartile, Max = maximum hourly particle counts.

Sensor ID	Variable	n	Mean	Median	Q1–Q3	Max
SPS30A		12670	86.2	49.8	25.3–109.2	946.0
SPS30B	PM_{10} count	9287	76.4	46.5	24.5–94.7	926.7
**Fidas***		12914	356.5	286.6	158.8–459.3	2415.5
SPS30A		12670	86.1	49.8	25.3–109.0	944.1
SPS30B	$PM_{2.5}$ count	9286	76.3	46.5	24.5–94.6	925.0
**Fidas**		12915	356.3	286.4	158.6–459.0	2415.4

* Specific episodes with high PM_{10} concentrations (described in Table 1) were excluded from these statistics.

Table S10. Comparison of PM_{10} and $PM_{2.5}$ particle counts measured by SPS30 sensors and the reference EM Fidas monitor. Performance metrics derived from simple linear regression include the coefficient of determination (R^2), regression slope and intercept, agreement between paired observations is additionally described by root mean squared error (RMSE), mean absolute error (MAE), bias and the number of valid observations (n).

Sensor	n	R^2	RMSE	MAE	Bias	Slope	Intercept
PM_{10} count SPS30A	12623	0.86	341.79	273.29	-273.29	0.31	-24.12
PM_{10} count SPS30B	9213	0.77	280.10	235.43	-235.28	0.33	-26.45
$PM_{2.5}$ count SPS30A	12624	0.86	341.79	273.26	-273.26	0.31	-24.05
$PM_{2.5}$ count SPS30B	9214	0.77	280.04	235.36	-235.20	0.33	-26.37

Table S11. Results of main three models tested for sensor performance in case of PM_{10} and $PM_{2.5}$ particle count measurement. SPR = Sensor Performance Ratio, season (cold or warm), RH = relative humidity (%), T = temperature (°C), DPDc = mean-centred Dew Point Deficit (°C). Model results: R^2 = coefficient of determination, AIC = Akaike Information Criterion, Adj. R^2 = adjusted coefficient of determination.

Model ID	Model despcription	Sensor ID	Model results		
			R^2	AIC	Adj. R^2
M1	PM_{10} count SPR ~ season	SPS30 A	0.41	-32284.3	0.41
		SPS30 B	0.37	-20034.0	0.37
M2	PM_{10} count SPR ~ season + RH * T	SPS30 A	**0.61**	-37464.0	0.61
		SPS30 B	**0.54**	-23003.7	0.54
M3	PM_{10} count SPR ~ season + DPDc + $DPDc^2$	SPS30 A	**0.58**	-36461.0	0.56
		SPS30 B	**0.52**	-23003.7	0.52

M4	$PM_{2.5}$ count SPR ~ season	SPS30 A	0.41	-32465.3	0.41
		SPS30 B	0.37	-20332.2	0.37
M5	$PM_{2.5}$ count SPR ~ season + RH * T	SPS30 A	**0.61**	-37666.2	0.61
		SPS30 B	**0.55**	-23375.7	0.55
M6	$PM_{2.5}$ count SPR ~ season + DPDc + DPDc²	SPS30 A	**0.58**	-36670.2	0.58
		SPS30 B	**0.53**	-23024.2	0.53

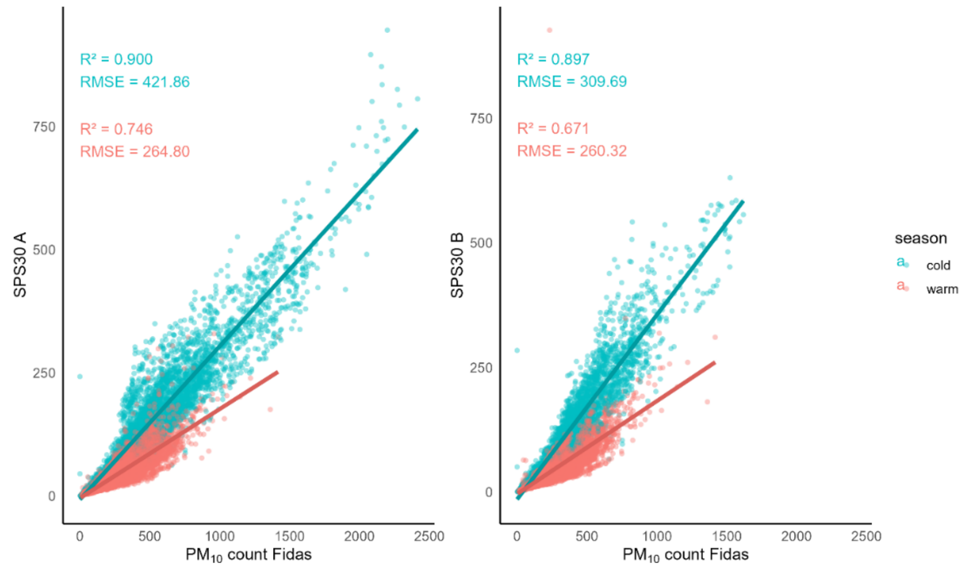

Figure S10. Comparison of PM_{10} particle count (particles·m⁻³) measured by SPS30 sensors (SPS30A and SPS30B) and the EM Fidas equivalent analyser during the cold and warm seasons in Tušimice between 1 February 2024 and 31 August 2025. Solid lines indicate season-specific linear regression fits (blue: cold season; red: warm season). Corresponding coefficients of determination (R^2) and root mean square errors (RMSE) are provided for each season.

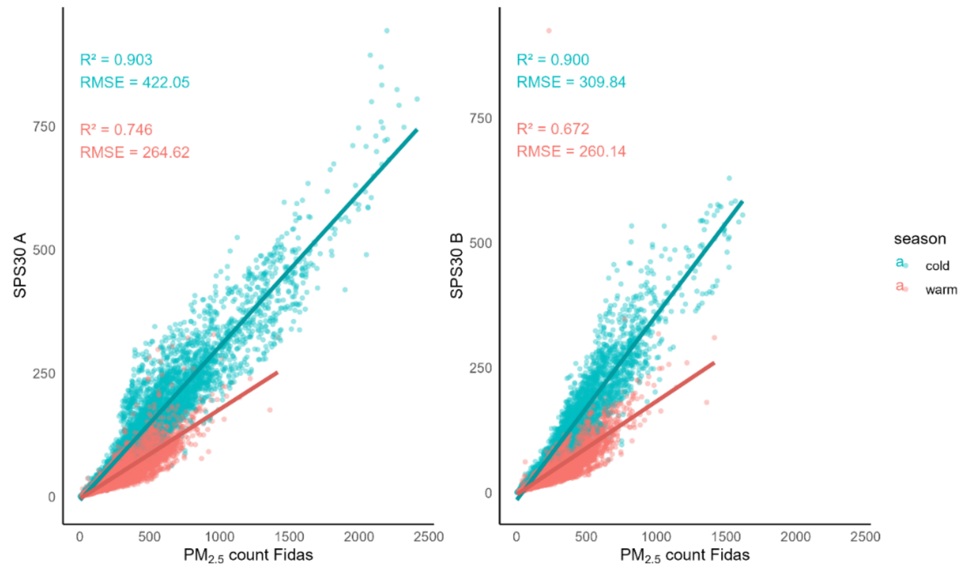

Figure S11. Comparison of $PM_{2.5}$ particle count (particles·m⁻³) measured by two SPS30 sensors (SPS30A and SPS30B) and the EM Fidas equivalent analyser during the cold and warm seasons in Tušimice between 1 February 2024 and 31 August 2025. Solid lines indicate season-specific linear regression fits (blue: cold season; red: warm season). Corresponding coefficients of determination (R^2) and root mean square errors (RMSE) are provided for each season.

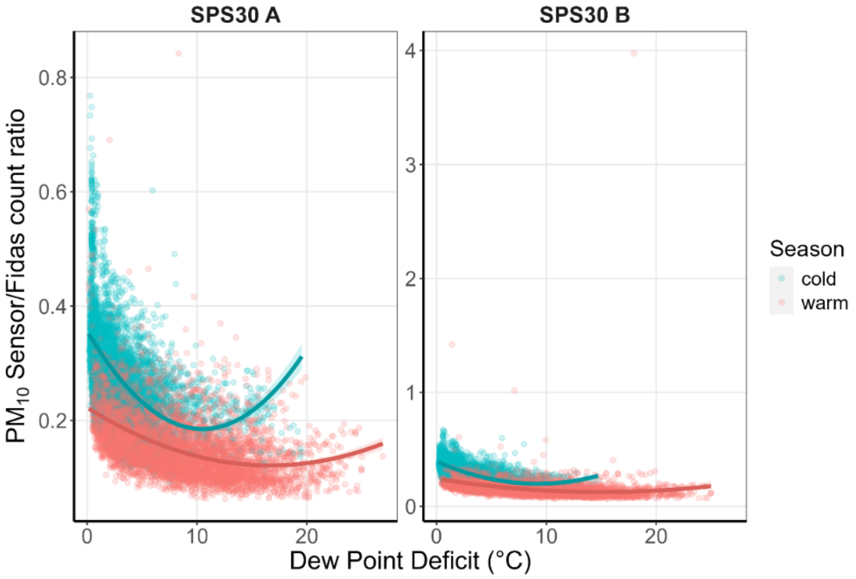

Figure S12. The relationship between the PM_{10} Sensor/Fidas count ratio (sensor performance ratio) and the dew point deficit (DPD in °C) during the cold season (blue) and the warm season (red). The nonlinear relationship results from the quadratic term in the DPD variable (model M3 in Table S11).

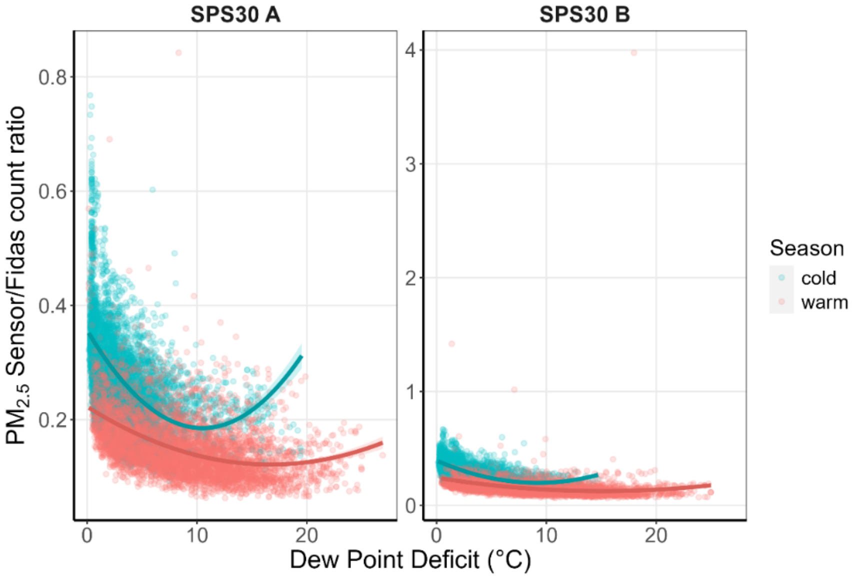

Figure S13. The relationship between the $PM_{2.5}$ Sensor/Fidas count ratio (sensor performance ratio) and the dew point deficit (DPD in °C) during the cold season (blue) and the warm season (red). The nonlinear relationship results from the quadratic term in the DPD variable (model M6 in Table S11).

Table S12. Performance of the main MARS correction models for PM_{10} measurement evaluated using the two-month training dataset (1 February–30 March 2024). Model performance is expressed by training R^2 and GCV error, while model complexity is represented by the number of terms in the final model.

Model ID	Model description	Sensor	Training R^2	GCV	Number of terms
MC1	PM_{10} ~ sensor + T+ RH	SEN54A	0.82	43.65	8
		SEN54B	0.82	41.74	8
		SPS30A	0.83	41.1	8
		SPS30B	0.83	41.03	8
MC2	PM_{10} ~ sensor + T * RH	SEN54A	0.83	41.67	12
		SEN54B	0.83	40.36	14
		SPS30A	0.84	38.9	15
		SPS30B	0.84	39.65	14
MC3	PM_{10} ~ sensor + DPD	SEN54A	0.81	44.48	7
		SEN54B	0.82	42.32	7
		SPS30A	0.82	42.33	7
		SPS30B	0.83	41.82	7
MC4	PM_{10} ~ sensor PM_1 count + DPD	SPS30A	0.84	38.81	5
		SPS30B	0.84	39.15	5

Table S13. Performance of the main MARS correction models for $PM_{2.5}$ measurement evaluated using the two-month training dataset (1 February–30 March 2024). Model performance is expressed by training R^2 and GCV error, while model complexity is represented by the number of terms in the final model.

Model ID	Model description	Sensor	Training R^2	GCV	Number of terms
MC1	$PM_{2.5}$ ~ sensor + T+ RH	SEN54A	0.94	8.75	6
		SEN54B	0.94	8.71	6
		SPS30A	0.95	7.35	7
		SPS30B	0.95	8.5	7
MC2	$PM_{2.5}$ ~ sensor + T * RH	SEN54A	0.94	8.66	7
		SEN54B	0.94	8.58	6
		SPS30A	0.95	7.2	8
		SPS30B	0.95	8.31	8
MC3	$PM_{2.5}$ ~ sensor + DPD	SEN54A	0.94	8.77	6
		SEN54B	0.94	8.68	5
		SPS30A	0.95	7.45	7
		SPS30B	0.95	8.49	7
MC4	$PM_{2.5}$ ~ sensor PM_1 count + DPD	SPS30A	0.96	6.63	4
		SPS30B	0.95	7.01	4

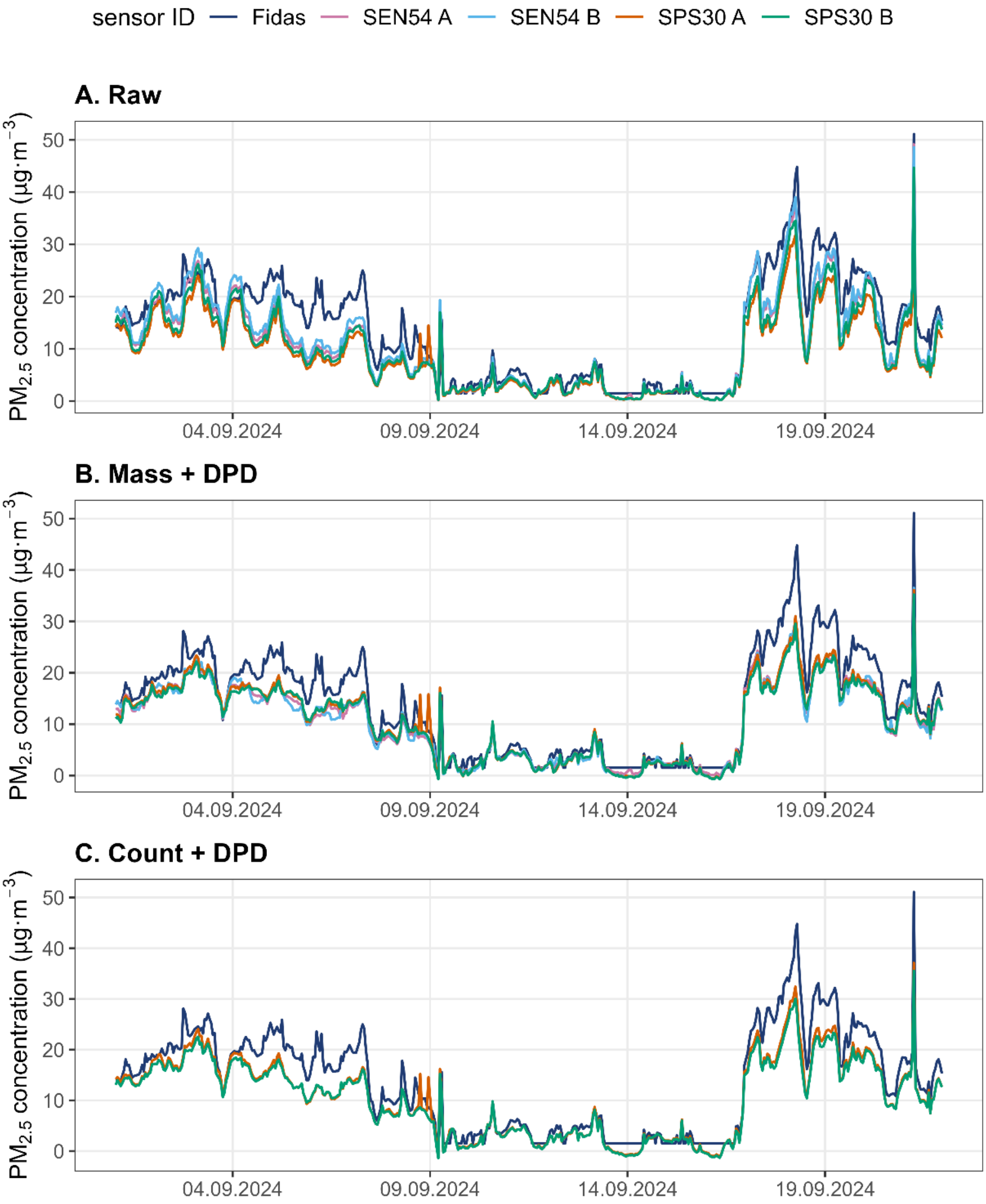

Figure S14. Representative time series of $PM_{2.5}$ concentrations measured by different sensors and the reference Fidas measurement during a warm-season episode (1–21 September 2024). Panel A shows raw sensor measurements, panel B concentrations corrected using sensor-reported PM mass concentration and dew point deficit (Mass + DPD), and panel C concentrations corrected using particle-count variables combined with dew point deficit (Count + DPD). Corrected concentrations were obtained using MARS models developed from a two-month training dataset (1 February–30 March 2024).

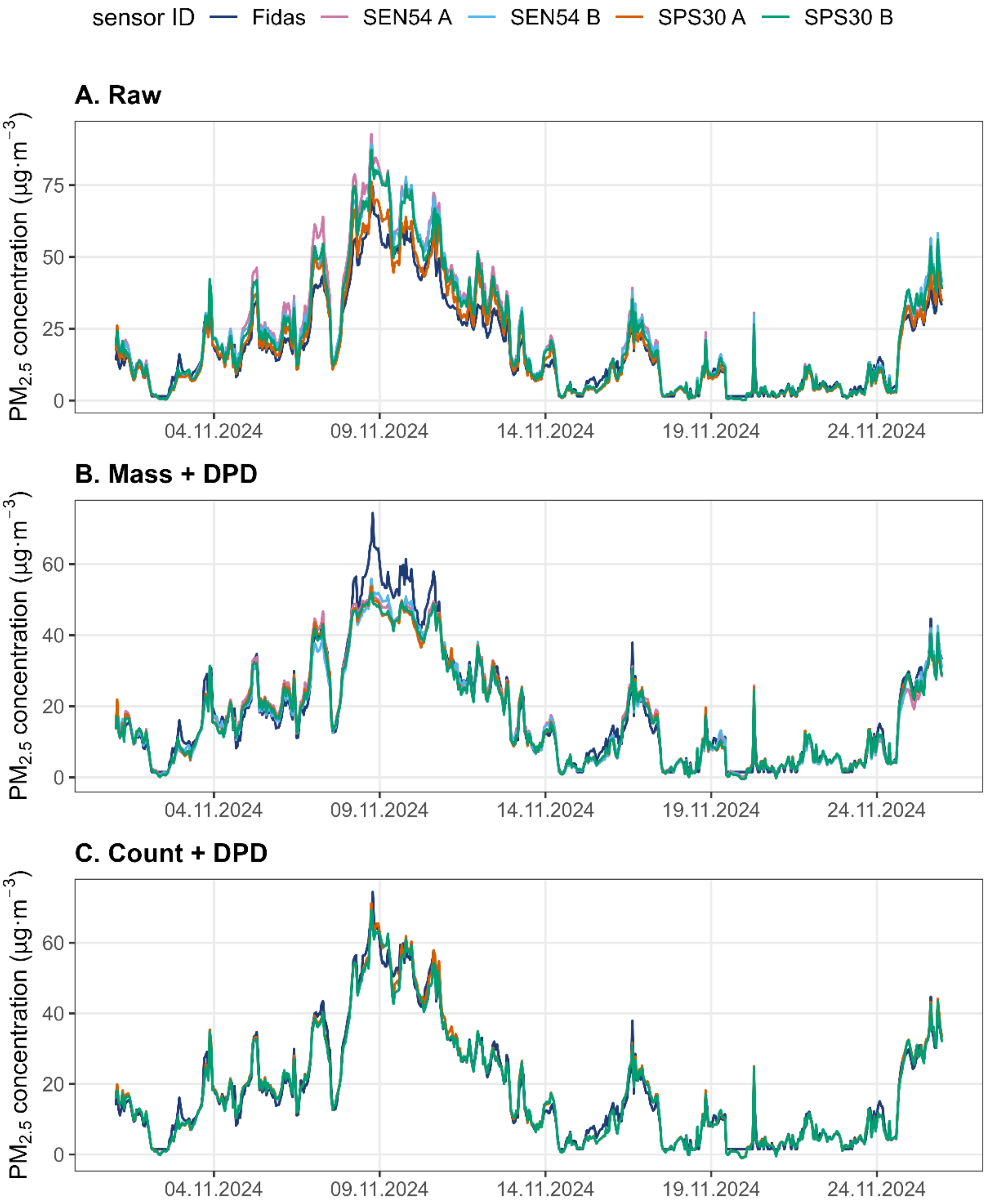

Figure S15. Representative time series of $PM_{2.5}$ concentrations measured by different sensors and the reference Fidas measurement during a cold-season episode (1–25 November 2024). Panel A shows raw sensor measurements, panel B concentrations corrected using sensor-reported PM mass concentration and dew point deficit (Mass + DPD), and panel C concentrations corrected using particle-count variables combined with dew point deficit (Count + DPD). Corrected concentrations were obtained using MARS models developed from a two-month training dataset (1 February–30 March 2024).

Table S14. Performance of the alternatively tested MARS correction models for PM_{10} measurement evaluated using the six-month training dataset (1 February–31 July 2024). Model performance is expressed by training R^2 and GCV error, while model complexity is represented by the number of terms in the final model.

Model ID	Model description	Sensor	Training R^2	GCV	Number of terms
CM1e	PM_{10} Fidas ~ PM_{10} sensor + DPD	SEN54A	0.67	45.14	6
		SEN54B	0.68	44.97	6
		SPS30A	0.69	42.33	6
		SPS30B	0.69	43.05	6
CM2e	PM_{10} Fidas ~ PM_{10} sensor + DPD + season	SEN54A	0.69	42.92	8
		SEN54B	0.69	42.95	7
		SPS30A	0.71	40.4	7
		SPS30B	0.7	40.68	7
CM3e	PM_{10} Fidas ~ PM_{10} sensor + DPD + hour	SEN54A	0.69	43.5	13
		SEN54B	0.69	43.26	12
		SPS30A	0.7	41.08	10
		SPS30B	0.7	41.81	10
CM4e	PM_{10} Fidas ~ PM_{10} sensor + DPD + season + hour	SEN54A	0.7	41.79	13
		SEN54B	0.7	41.48	13
		SPS30A	0.72	38.61	14
		SPS30B	0.72	38.92	14
CM5e	PM_{10} Fidas ~ PM_{10} sensor + DPD + season + hour + interaction	SEN54A	0.71	41	17
		SEN54B	0.71	40.59	17
		SPS30A	0.73	37.58	19
		SPS30B	0.73	37.7	16

Table S15. Performance of the alternatively tested MARS correction models for $PM_{2.5}$ measurement evaluated using the six-month training dataset (1 February–31 July 2024). Model performance is expressed by training R^2 and GCV error, while model complexity is represented by the number of terms in the final model.

Model ID	Model description	Sensor	Training R^2	GCV	Number of terms
CM1e	$PM_{2.5}$ Fidas ~ $PM_{2.5}$ sensor + DPD	SEN54A	0.93	5.4	6
		SEN54B	0.92	6.47	6
		SPS30A	0.94	4.61	6
		SPS30B	0.93	5.17	7
CM2e	$PM_{2.5}$ Fidas ~ $PM_{2.5}$ sensor + DPD + season	SEN54A	0.93	5.26	7
		SEN54B	0.92	6.37	7
		SPS30A	0.94	4.52	7
		SPS30B	0.94	4.98	8
CM3e	$PM_{2.5}$ Fidas ~ $PM_{2.5}$ sensor + DPD + hour	SEN54A	0.93	5.4	6
		SEN54B	0.92	6.47	6
		SPS30A	0.94	4.61	6
		SPS30B	0.93	5.18	7
CM4e	$PM_{2.5}$ Fidas ~ $PM_{2.5}$ sensor + DPD + season + hour	SEN54A	0.93	5.26	7
		SEN54B	0.92	6.37	7
		SPS30A	0.94	4.52	7

		SPS30B	0.94	4.98	8
CM5e	$PM_{2.5}$ Fidas ~ $PM_{2.5}$ sensor + DPD + season + hour + interaction	SEN54A	0.94	4.98	12
		SEN54B	0.94	5.25	17
		SPS30A	0.95	3.9	11
		SPS30B	0.94	4.38	12

Table S16. Performance of PM_{10} alternative correction models on an independent testing dataset (extended-training scenario: six-month training dataset). Results are shown for the tested correction models and individual sensors, including the number of valid observations (n), the coefficient of determination (R^2), root mean square error (RMSE), mean absolute error (MAE), bias, and the slope and intercept of the linear regression between corrected sensor concentrations and EM Fidas measurements.

**Model ID**	**Model predictors**	**Sensor**	**n**	**R^2**	**RMSE**	**MAE**	**Bias**	**Slope**	**Intercept**
CM0e	PM_{10} sensor	SEN54A	9084	0.64	12.65	9.10	-3.41	1.06	-4.65
		SEN54B	9084	0.70	8.41	5.69	-0.57	0.65	6.58
		SPS30A	9084	0.72	8.18	5.47	-0.77	0.67	5.88
		SPS30B	9084	0.73	8.06	5.40	-0.97	0.65	6.04
CM1e	PM_{10} sensor + DPD	SEN54A	9084	0.74	7.88	5.25	-1.12	0.68	5.41
		SEN54B	9084	0.75	7.64	5.12	-0.89	0.71	5.09
		SPS30A	9115	0.63	12.02	8.37	-3.99	0.98	-3.61
		SPS30B	9115	0.71	8.29	5.46	-0.54	0.66	6.40
CM2e	PM_{10} sensor + DPD + season	SEN54A	9115	0.72	8.05	5.24	-0.74	0.68	5.71
		SEN54B	9115	0.72	8.12	5.36	-0.63	0.67	6.13
		SPS30A	9115	0.73	7.96	5.22	-0.75	0.69	5.58
		SPS30B	9115	0.70	8.35	5.17	-0.73	0.69	5.51
CM3e	PM_{10} sensor + DPD + hour	SEN54A	9080	0.65	11.64	8.51	-6.6	0.83	-3.32
		SEN54B	9080	0.70	8.34	5.62	-0.11	0.67	6.55
		SPS30A	9080	0.72	8.08	5.35	-0.33	0.70	5.70
		SPS30B	9080	0.71	8.15	5.46	-0.20	0.68	6.21
CM4e	PM_{10} sensor + DPD + season + hour	SEN54A	9080	0.75	7.65	5.11	-0.70	0.71	5.25
		SEN54B	9080	0.78	7.12	4.77	-0.37	0.77	4.30
		SPS30A	5672	0.53	11.13	8.1	-6.14	0.74	-1.66
		SPS30B	5672	0.65	7.31	4.76	-0.42	0.65	5.68
CM5e	PM_{10} sensor + DPD + season + hour + interaction	SEN54A	5672	0.69	6.98	4.53	-0.67	0.70	4.66
		SEN54B	5672	0.67	7.17	4.67	-0.59	0.66	5.31
		SPS30A	5672	0.70	6.86	4.45	-1.11	0.68	4.49
		SPS30B	5672	0.65	7.38	4.31	-0.95	0.67	4.77

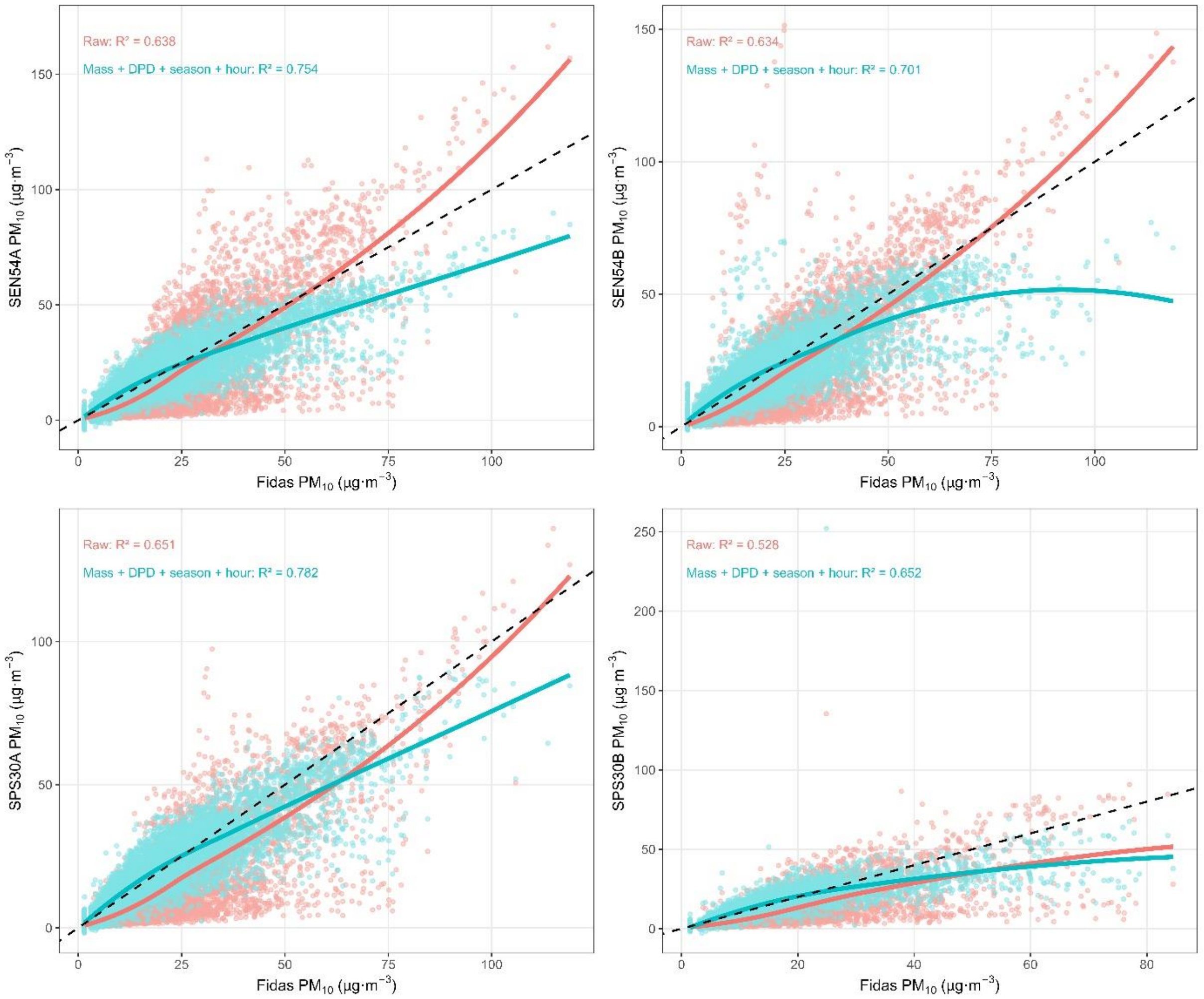

Figure S16. Relationship between PM_{10} concentrations measured by individual sensors (y-axis) and the equivalent monitor Fidas (x-axis). Raw sensor measurements (red) are compared with concentrations corrected using sensor-reported PM mass concentration, dew point deficit, season and hour of the day, including pairwise interactions (light blue). Correction models were developed using a six-month training dataset (1 February–31 July 2024; see CM5e in Table S14), whereas model performance was evaluated using an independent testing dataset (1 August 2024–31 August 2025). R^2 values from linear regression are shown in each panel. The dashed black line represents the 1:1 relationship. LOESS smoothing curves (span = 0.90) are included to illustrate trends in sensor response.

Table S17. Performance of $PM_{2.5}$ alternative correction models on an independent testing dataset (extended-training scenario: six-month training dataset). Results are shown for the tested correction models and individual sensors, including the number of valid observations (n), the coefficient of determination (R^2), root mean square error (RMSE), mean absolute error (MAE), bias, and the slope and intercept of the linear regression between corrected sensor concentrations and EM Fidas measurements.

Model ID	Model predictors	Sensor	n	R^2	RMSE	MAE	Bias	Slope	Intercept
CM0e	$PM_{2.5}$ sensor	SEN54A	9036	0.93	5.69	3.71	0.83	1.27	-2.97

		SEN54B	9115	0.85	7.75	3.91	1.40	1.27	-2.41
		SPS30A	9080	0.93	4.39	3.11	-0.81	1.12	-2.49
		SPS30B	5672	0.88	4.60	3.11	-0.48	1.19	-2.70
CM1e	$PM_{2.5}$ sensor + DPD	SEN54A	9036	0.93	3.60	2.12	-0.49	0.84	1.70
		SEN54B	9115	0.89	4.20	2.14	-0.37	0.84	1.86
		SPS30A	9080	0.93	3.38	2.05	-0.06	0.88	1.65
		SPS30B	5672	0.93	2.59	1.63	-0.45	0.89	0.86
CM2e	$PM_{2.5}$ sensor + DPD + season	SEN54A	9036	0.93	3.50	2.06	-0.56	0.85	1.54
		SEN54B	9115	0.89	4.17	2.09	-0.42	0.84	1.75
		SPS30A	9080	0.93	3.35	1.98	-0.12	0.88	1.53
		SPS30B	5672	0.93	2.50	1.55	-0.54	0.89	0.69
CM3e	$PM_{2.5}$ sensor + DPD + hour	SEN54A	9036	0.93	3.52	2.12	-0.49	0.85	1.67
		SEN54B	9115	0.89	4.22	2.14	-0.36	0.84	1.85
		SPS30A	9080	0.93	3.39	2.05	-0.06	0.88	1.65
		SPS30B	5672	0.93	2.60	1.63	-0.45	0.88	0.87
CM4e	$PM_{2.5}$ sensor + DPD + season + hour	SEN54A	9036	0.93	3.50	2.06	-0.56	0.85	1.54
		SEN54B	9115	0.90	4.16	2.09	-0.43	0.84	1.77
		SPS30A	9080	0.93	3.35	1.98	-0.12	0.88	1.53
		SPS30B	5672	0.93	2.50	1.55	-0.54	0.89	0.69
CM5e	$PM_{2.5}$ sensor + DPD + season + hour + interaction	SEN54A	9036	0.95	3.13	1.94	-0.44	0.88	1.30
		SEN54B	9115	0.84	5.10	2.20	-0.31	0.85	1.72
		SPS30A	9080	0.96	2.70	1.72	-0.07	0.92	1.02
		SPS30B	5672	0.87	3.46	1.50	-0.50	0.87	0.99

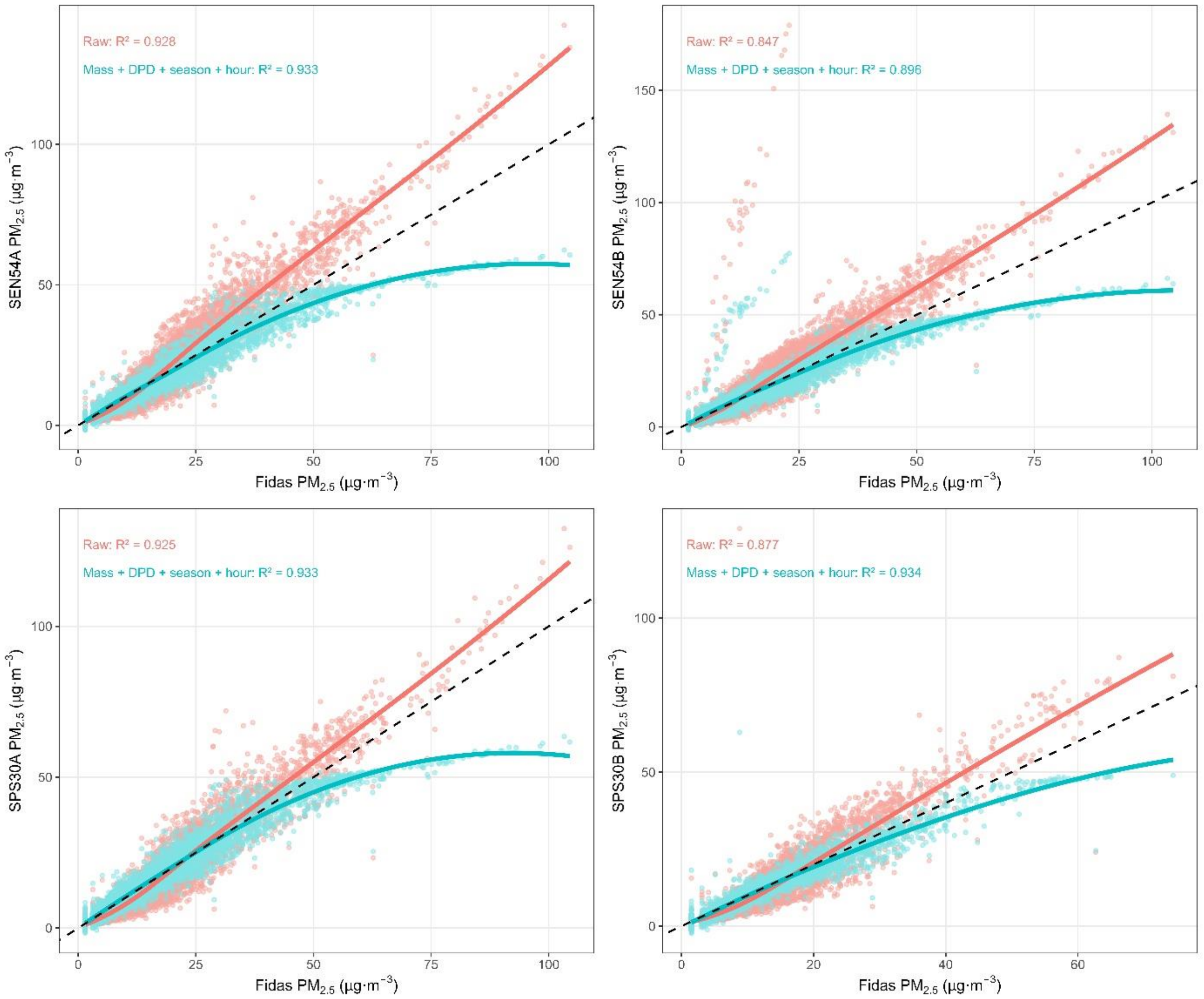

Figure S17. Relationship between $PM_{2.5}$ concentrations measured by individual sensors (y-axis) and the equivalent- monitor Fidas (x-axis). Raw sensor measurements (red) are compared with concentrations corrected using sensor-reported PM mass concentration, dew point deficit, season and hour of the day, including pairwise interactions (light blue). Correction models were developed using a six-month training dataset (1 February–31 July 2024; see CM5e in Table S15), whereas model performance was evaluated using an independent testing dataset (1 August 2024–31 August 2025). R^2 values from linear regression are shown in each panel. The dashed black line represents the 1:1 relationship. LOESS smoothing curves (span = 0.90) are included to illustrate trends in sensor response.

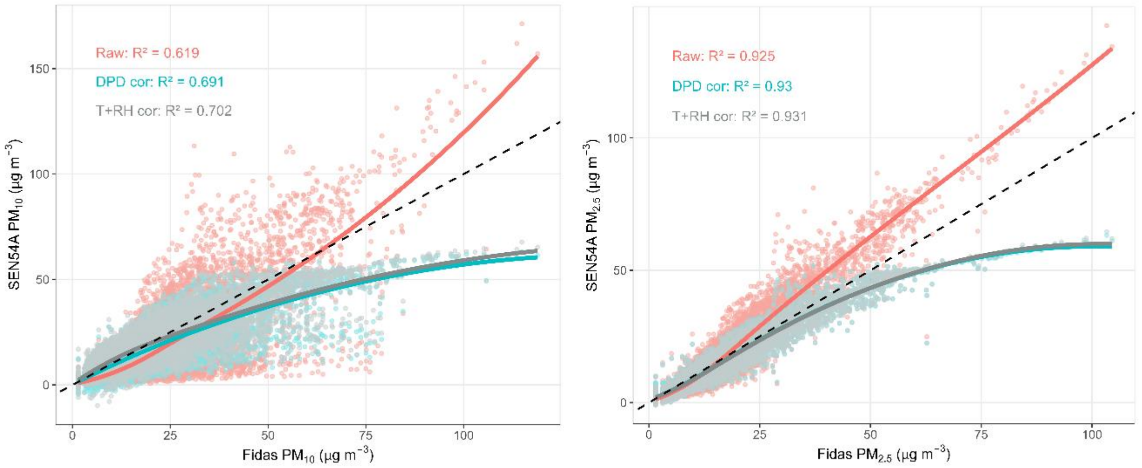

Figure S18. Example of relationship between PM_{10} and $PM_{2.5}$ concentrations measured by sensor SEN54A (y-axis) and the equivalent monitor Fidas (x-axis). Raw sensor measurements (red) are compared with concentrations corrected using sensor-reported PM mass concentration and dew point deficit (DPD cor; light blue) and with sensor-reported PM mass concentration combined with temperature and relative humidity (T + RH cor; grey). Correction models were developed using a two-month training dataset (1 February–30 March 2024), whereas model performance was evaluated using an independent testing dataset (31 March 2024–31 August 2025). R^2 values obtained from linear regression are shown in each panel. The dashed black line represents the 1:1 relationship. LOESS smoothing curves (span = 0.90) are included to illustrate trends in sensor response.

Table S18. Performance of PM_{10} measurements in two concentration ranges (≤ 50 and > 50 $\mu g \cdot m^{-3}$) before and after different corrections. Raw indicates original sensor data, mass T + RH indicates correction based on sensor-reported PM mass concentration and temperature and humidity, mass + T*RH indicates correction based on PM mass concentration and temperature and humidity (including interaction), count + DPD indicates correction based on particle-count variables and dew point deficit. Performance statistics were calculated using an independent testing dataset (31 March 2024–31 August 2025). Statistical metrics (R^2, RMSE, MAE, bias, regression slope, and intercept) were calculated relative to the reference Fidas monitor, n= number of valid observations.

**Sensor**	**Conc. range**	**n**	**R^2**	**RMSE**	**MAE**	**Bias**	**Slope**	**Intercept**
SEN54A PM_{10}	raw ≤ 50	10954	0.48	11.10	8.32	-4.67	0.93	-3.47
	mass T+RH ≤ 50	10954	0.63	6.50	4.77	-0.31	0.74	4.05
	mass T*RH ≤ 50	10954	0.38	11.10	8.45	4.67	0.72	9.33
	mass DPD ≤ 50	10954	0.62	7.14	5.07	-2.52	0.75	1.73
	raw > 50	536	0.24	26.90	21.93	0.38	1.26	-16.32
	mass T+RH > 50	536	0.14	21.77	17.80	-17.72	0.34	23.92
	mass T*RH > 50	536	0.13	20.07	16.23	-15.41	0.34	26.53
	mass DPD > 50	536	0.11	23.30	18.95	-18.90	0.32	23.76
SEN54B PM_{10}	raw ≤ 50	10902	0.47	10.81	7.81	-4.89	0.86	-2.47
	mass T+RH ≤ 50	10902	0.64	6.44	4.66	0.39	0.76	4.46
	mass T*RH ≤ 50	10902	0.42	10.45	7.85	4.52	0.73	9.02
	mass DPD ≤ 50	10902	0.62	7.04	4.86	-2.26	0.75	1.91
	raw > 50	536	0.24	24.11	19.01	-3.16	1.15	-12.42
	mass T+RH > 50	536	0.11	21.04	16.66	-16.57	0.29	28.15
	mass T*RH > 50	536	0.09	19.63	15.20	-14.21	0.28	31.43

	mass DPD > 50	536	0.08	22.58	17.64	-17.59	0.29	26.94
SPS30A PM_{10}	raw ≤ 50	10950	0.50	10.61	8.20	-6.83	0.73	-2.26
	mass T+RH ≤ 50	10950	0.65	6.40	4.85	0.69	0.78	4.35
	mass T*RH ≤ 50	10950	0.01	122.50	73.58	70.50	0.98	70.78
	mass DPD ≤ 50	10950	0.65	6.74	4.77	-1.84	0.79	1.65
	count DPD ≤ 50	10950	0.67	6.52	4.61	-1.49	0.83	1.33
	raw > 50	536	0.24	24.18	17.45	-13.05	0.98	-11.93
	mass T+RH > 50	536	0.12	20.60	16.42	-16.36	0.30	27.87
	mass T*RH > 50	536	0.00	55.24	28.79	6.69	0.02	68.74
	mass DPD > 50	536	0.08	22.39	17.77	-17.74	0.27	28.14
	count DPD > 50	536	0.29	19.62	14.63	-4.48	1.03	-6.37
SPS30B PM_{10}	raw ≤ 50	7910	0.45	10.20	7.92	-6.64	0.67	-1.52
	mass T+RH ≤ 50	7910	0.61	6.05	4.45	-0.01	0.71	4.60
	mass T*RH ≤ 50	7910	0.34	11.07	8.62	5.06	0.70	9.68
	mass DPD ≤ 50	7910	0.61	6.69	4.66	-2.87	0.68	2.08
	count DPD ≤ 50	7910	0.63	6.47	4.41	-2.66	0.70	2.01
	raw > 50	168	0.01	32.80	26.40	-19.60	0.23	27.20
	mass T+RH > 50	168	0.00	25.61	22.07	-22.00	0.08	34.05
	mass T*RH > 50	168	0.00	23.06	19.48	-19.29	0.07	36.99
	mass DPD > 50	168	0.00	28.45	24.37	-24.36	0.03	34.33
	count DPD > 50	168	0.01	27.34	21.17	-19.14	0.24	27.26

Table S19. Performance of $PM_{2.5}$ measurements in two concentration ranges (≤ 50 and > 50 $\mu g \cdot m^{-3}$) before and after different corrections. Raw indicates original sensor data, mass T + RH indicates correction based on sensor-reported PM mass concentration and temperature and humidity, mass + T*RH indicates correction based on PM mass concentration and temperature and humidity (including interaction), count + DPD indicates correction based on particle-count variables and dew point deficit. Performance statistics were calculated using an independent testing dataset (31 March 2024–31 August 2025). Statistical metrics (R^2, RMSE, MAE, bias, regression slope, and intercept) were calculated relative to the reference Fidas monitor, n= number of valid observations.

**Sensor**	**Conc. range**	**n**	**R^2**	**RMSE**	**MAE**	**Bias**	**Slope**	**Intercept**
SEN54A $PM_{2.5}$	raw ≤ 50	11197	0.89	4.71	3.17	-0.01	1.26	-3.00
	mass T+RH ≤ 50	11197	0.92	2.75	1.98	-1.02	0.93	-0.18
	mass T*RH ≤ 50	11197	0.93	2.44	1.74	0.02	0.91	1.10
	mass DPD ≤ 50	11197	0.92	2.70	1.95	-0.87	0.93	-0.02
	raw > 50	245	0.77	16.32	14.46	13.34	1.38	-9.68
	mass T+RH > 50	245	0.62	14.04	11.71	-11.71	0.37	26.66
	mass T*RH > 50	245	0.74	13.49	11.66	-11.65	0.45	21.66
	mass DPD > 50	245	0.61	14.28	11.90	-11.89	0.35	27.45
SEN54B $PM_{2.5}$	raw ≤ 50	11193	0.78	6.73	3.32	0.52	1.26	-2.53
	mass T+RH ≤ 50	11193	0.85	3.83	2.06	-0.56	0.95	-0.04
	mass T*RH ≤ 50	11193	0.86	3.54	1.89	0.49	0.92	1.46
	mass DPD ≤ 50	11193	0.86	3.67	1.96	-0.79	0.94	-0.11
	raw > 50	245	0.79	16.48	14.77	13.99	1.36	-7.61
	mass T+RH > 50	245	0.79	11.73	10.15	-10.14	0.55	16.79
	mass T*RH > 50	245	0.82	11.64	10.14	-10.13	0.56	16.54
	mass DPD > 50	245	0.80	11.75	10.22	-10.21	0.56	16.49

SPS30A $PM_{2.5}$	raw ≤ 50	11241	0.89	3.98	2.90	-1.30	1.10	-2.48
	mass T+RH ≤ 50	11241	0.93	2.55	1.82	-0.60	0.96	-0.19
	mass T*RH ≤ 50	11241	0.93	2.52	1.91	0.74	0.94	1.48
	mass DPD ≤ 50	11241	0.93	2.48	1.76	-0.40	0.97	-0.05
	count DPD ≤ 50	11241	0.94	2.50	1.74	-0.35	1.04	-0.82
	raw > 50	245	0.76	9.70	7.81	4.96	1.26	-10.70
	mass T+RH > 50	245	0.75	12.00	10.09	-10.07	0.48	21.18
	mass T*RH > 50	245	0.76	12.02	10.04	-10.02	0.47	22.23
	mass DPD > 50	245	0.75	12.52	10.52	-10.50	0.45	22.96
	count DPD > 50	245	0.81	7.43	5.80	3.14	1.19	-8.16
SPS30B $PM_{2.5}$	raw ≤ 50	8025	0.84	4.05	2.84	-1.05	1.15	-2.52
	mass T+RH ≤ 50	8025	0.91	2.58	1.83	-1.22	0.89	-0.09
	mass T*RH ≤ 50	8025	0.91	2.33	1.71	0.46	0.88	1.69
	mass DPD ≤ 50	8025	0.91	2.49	1.75	-1.02	0.89	0.08
	count DPD ≤ 50	8025	0.91	2.54	1.69	-1.10	0.93	-0.41
	raw > 50	53	0.14	14.88	12.97	10.42	0.87	17.74
	mass T+RH > 50	53	0.05	12.06	10.58	-10.58	0.20	34.95
	mass T*RH > 50	53	0.09	11.84	10.62	-10.62	0.22	33.79
	mass DPD > 50	53	0.03	12.19	10.68	-10.68	0.16	37.30
	count DPD > 50	53	0.17	7.84	4.93	-2.79	0.66	16.37
